# Infrared spectroscopy reveals metal-independent carbonic anhydrase activity in crotonyl-CoA carboxylase/reductase†

**DOI:** 10.1039/d3sc04208a

**Published:** 2024-02-29

**Authors:** Aharon Gomez, Matthias Tinzl, Gabriele Stoffel, Hendrik Westedt, Helmut Grubmüller, Tobias J. Erb, Esteban Vöhringer-Martinez, Sven T. Stripp

**Affiliations:** a Departamento de Físico Química, Facultad de Ciencias Químicas, Universidad de Concepción Concepción Chile evohringer@udec.cl; b Department of Biochemistry and Synthetic Metabolism, Max-Planck-Institute for Terrestrial Microbiology Karl-von-Frisch-Str. 10 D-35043 Marburg Germany; c Department of Theoretical and Computational Biophysics, Max-Planck-Institute for Multidisciplinary Sciences Am Fassberg 11 37077 Göttingen Germany; d Center for Synthetic Microbiology (SYNMIKRO) Germany; e Freie Universität Berlin, Experimental Molecular Biophysics Arnimallee 14 14195 Berlin Germany; f Technische Universität Berlin, Division of Physical Chemistry Strasse des 17. Juni 124 10623 Berlin Germany s.stripp@tu-berlin.de

## Abstract

The conversion of CO_2_ by enzymes such as carbonic anhydrase or carboxylases plays a crucial role in many biological processes. However, *in situ* methods following the microscopic details of CO_2_ conversion at the active site are limited. Here, we used infrared spectroscopy to study the interaction of CO_2_, water, bicarbonate, and other reactants with β-carbonic anhydrase from *Escherichia coli* (*Ec*CA) and crotonyl-CoA carboxylase/reductase from *Kitasatospora setae* (*Ks*Ccr), two of the fastest CO_2_-converting enzymes in nature. Our data reveal that *Ks*Ccr possesses a so far unknown metal-independent CA-like activity. Site-directed mutagenesis of conserved active site residues combined with molecular dynamics simulations tracing CO_2_ distributions in the active site of *Ks*CCr identify an ‘activated’ water molecule forming the hydroxyl anion that attacks CO_2_ and yields bicarbonate (HCO_3_^−^). Computer simulations also explain why substrate binding inhibits the anhydrase activity. Altogether, we demonstrate how *in situ* infrared spectroscopy combined with molecular dynamics simulations provides a simple yet powerful new approach to investigate the atomistic reaction mechanisms of different enzymes with CO_2_.

## Abstract 2

Using infrared spectroscopy and molecular simulations we show that the fastest CO_2_-fixing enzyme found in nature has an unknown anhydrase activity in absence of substrate.

## Graphical abstract

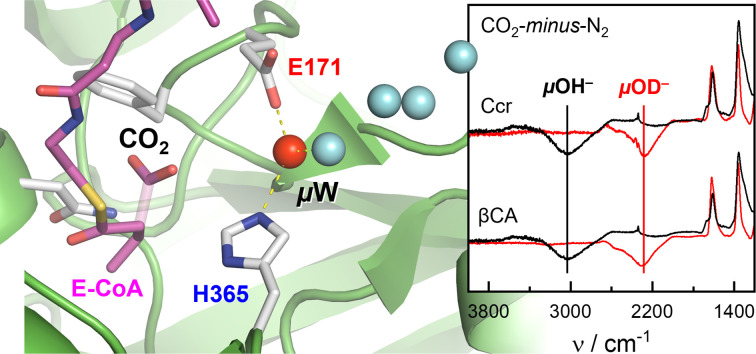

## Introduction

Developing catalytic strategies for the capture and conversion of carbon dioxide (CO_2_) is key to increased mitigation, utilization, and sequestration of this critical greenhouse gas. While still being a challenge for synthetic chemistry enzymes provide a natural blueprint for efficient CO_2_-converting catalysts.^1^ Several enzymes are known that interact with CO_2_ and/or bicarbonate (HCO_3_^−^) during catalysis, in particular carbonic anhydrases (CAs) and carboxylases.

**Scheme 1:**
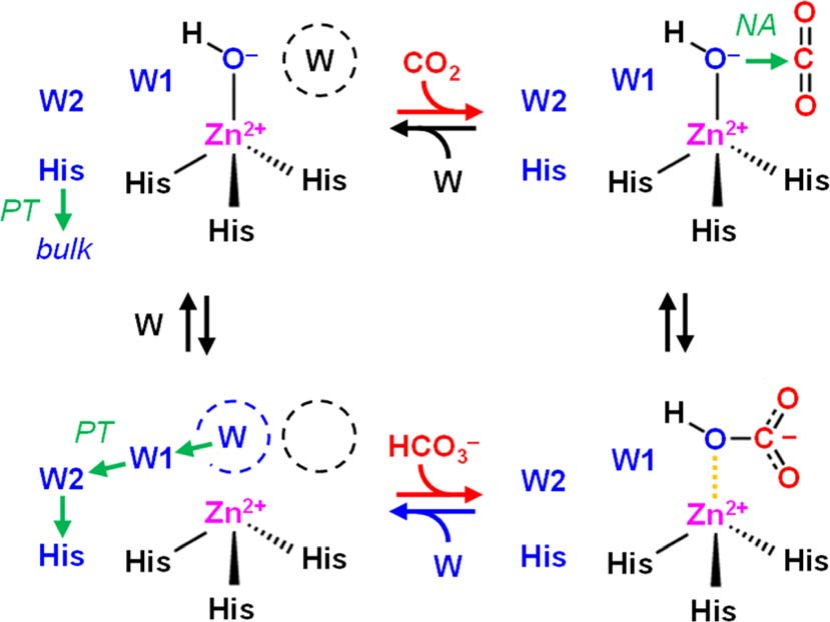
Mechanism of CO_2_ hydration in carbonic anhydrase. Top row, left to right: CO_2_ enters the active site and replaces a water molecule (W, black) near the zinc cation (Zn^2+^). In the next step, CO_2_ is converted to HCO_3_^−^ upon a nucleophilic attack (NA) of the zinc-bound hydroxide (Zn^2+^–OH^−^). Bottom row, right to left: HCO_3_^−^ is replaced by another water molecule (W, blue), the latter which is activated to OH^−^ upon proton transfer (PT) *via* oriented water molecules W1 and W2 and a conserved histidine (His, blue). In the last step, this histidine changes its orientation to release the proton into bulk solvent, and water re-binds (W, black) near the active site.

CAs catalyze the reversible conversion of CO_2_, H_2_O, and bicarbonate (HCO_3_^−^) with rate enhancements of close to 8 × 10^6^ compared to the reaction in aqueous solution (eqn (1)). This makes them one of the most effective CO_2_-converting catalysts in nature.^2^ CAs are present in all three domains of life and have been classified in eight families.^3^ Almost all known CAs feature a zinc cation (Zn^2+^) as active site cofactor,^4^ which plays a central role in catalysis as Zn^2+^ coordinates a hydroxide anion (OH^−^) that attacks CO_2_ as a nucleophile to form HCO_3_^−^ (Scheme 1). The OH^−^ species itself is generated through proton abstraction from a zinc-bound water molecule to a nearby base, which is usually a histidine.^5–8^

1 CO_2_ + 2H_2_O ↔ HCO_3_^−^ + H_3_O^+^

**Fig. 1:**
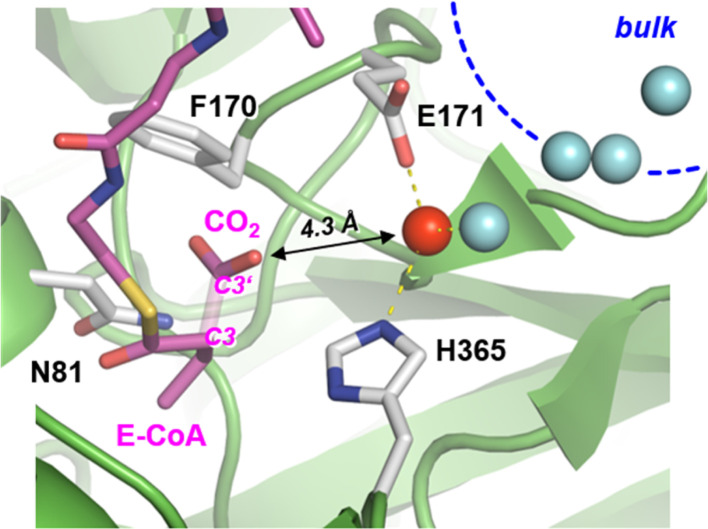
Active site of crotonyl CoA carboxylase/reductase. Crystal structure of *Ks*Ccr in complex with reaction product ethylmalonyl coenzyme A, E-CoA (PDB ID 6OWE). The bond between C3 and C3′ of E-CoA is drawn translucent to emphasize the CO_2_ binding site. *Ks*Ccr active site residues F170 and N81 interact with E-CoA while H365 and E171 coordinate a ‘bridging’ water molecule, *μ*W (red sphere, distance to H365 and E171 each 2.8 Å). A local water cluster connects the active site with bulk solvent (blue spheres, shortest distance to *μ*W 2.9 Å).

Carboxylases catalyze the addition of CO_2_ to an acceptor substrate with the family of enoyl-CoA carboxylases/reductases (ECRs) encompassing some of the most efficient CO_2_-fixing enzymes found in nature.^9^ ECRs catalyze the reductive carboxylation of α,β-unsaturated enoyl-CoAs with the reduced form of nicotinamide adenine dinucleotide phosphate (NADPH) as cofactor. Hydride transfer from NADPH to the enoyl-CoA substrate generates a reactive enolate species, which acts as a nucleophile that attacks a CO_2_ molecule bound at the active site.^10,11^ At the example of crotonyl-CoA carboxylase/reductase from *Kitasatospora setae* (*Ks*Ccr), Fig. 1 illustrates how CO_2_-binding is achieved through four amino acid residues and one conserved water molecule that is coordinated by an aspartate E171 and a histidine H365 (*μ*W).^12^

All molecular species involved in the above described CO_2_-conversions (H_2_O, CO_2_, HCO_3_^−^) show characteristic absorbance between 4000–1000 cm^−1^, which makes them available to Fourier-transform infrared (FTIR) spectroscopy.^13–16^ In a protein sample, however, these signals are overlaid by the intense absorbance of bulk water and the amide bands of the protein backbone.^17^ This limitation can be overcome by FTIR difference spectroscopy, which provides the means to distinguish between protein sample background signals and the signature of a given reaction upon a specific trigger.^18^ We developed a FTIR difference spectroscopy-based setup in which catalysis can be triggered *via* the gas phase.^19^ Compared to the conventional transmission configuration a protein film is formed on top of the silicon crystal of an attenuated total reflection (ATR) optical cell,^20^ which makes the protein amendable to changes in the gas phase, *e.g.*, by switching from a inert carrier gas (100% N_2_ or Ar, defining the background signal) to a ‘reactive’ gas mixture (see ESI† for further details). This specific design allows studying the reaction of CO_2_-converting enzymes providing the substrate (*i.e.,* CO_2_) *in situ* and thus the reaction trigger for these enzymes.^19^

Here, we applied *in situ* ATR FTIR spectroscopy to study the interaction of *Ks*Ccr with CO_2_. Our results show that the active site of *Ks*Ccr does not only bind CO_2_ but surprisingly possesses a so-far unknown, intrinsic CA-like activity, which enables the enzyme to catalyze the reversible interconversion of CO_2_, H_2_O, and HCO_3_^−^. Studying the reaction in absence or presence of substrates or inhibitors with wild-type and five active site variants, we identified key residues for the observed CA-like activity including a cluster of strongly hydrogen-bonded, ‘local’ water molecules. Moreover, computer simulations suggest that conformational dynamics and substrate binding in *Ks*Ccr modulate CO_2_ binding at the active site. Combining experiment and simulation, we propose a mechanism for the CA-like activity of *Ks*Ccr that involves an ‘activated’ water molecule, which is essential for CO_2_-binding during the CO_2_-fixation reaction of *Ks*Ccr but also serves as nucleophilic OH^−^ anion in the enzyme's CA-like reaction.

## Results and discussion

### Infrared signatures of anhydrase activity

First, we pipetted 1 μl *Ks*Ccr solution (200 μM protein in 25 mM Tris/HCl pH 7.5) on the ATR crystal of the FTIR spectrometer and monitored water evaporation under dry N_2_ gas *in situ*. Once sufficiently concentrated, we rehydrated the protein film under a stream of aerosol that was created by sending dry N_2_ gas (3 L min^−1^) through a wash bottle containing a dilute Tris/HCl buffer solution (1 mM, pH 7.5). Then, we added 10% CO_2_ to the N_2_ carrier gas for 50–100 s and recorded data to calculate a series of time-resolved *in situ* ATR FTIR difference spectra that result from the interaction of *Ks*Ccr with CO_2_ (Fig. S1†). In reference experiments with pure water and buffer solution, 25 mM Tris/HCl (pH 8) was found to be sufficiently concentrated preventing acidification in the presence of 10% CO_2_ (Fig. S2†).

**Fig. 2:**
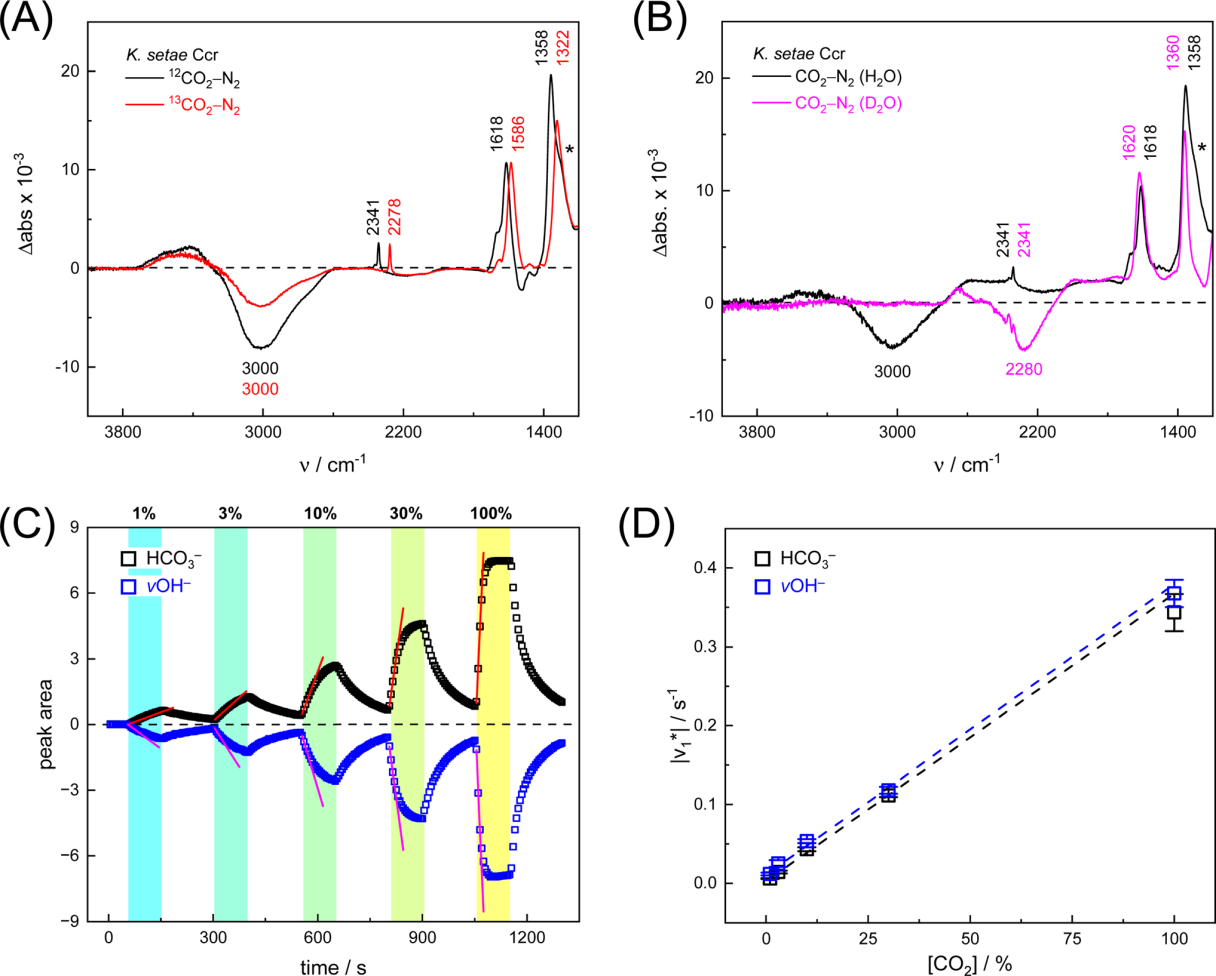
Infrared characterization of the reaction of *Ks*Ccr with CO_2_. (A) ‘CO_2_–N_2_’ ATR FTIR difference spectra for the reaction with ^12^CO_2_ (black) or ^13^CO_2_ (red). Positive signals are assigned to CO_2_ (2341 or 2278 cm^−1^) and HCO_3_^−^. The COH vibration (*v*_4_) appears as a shoulder at 1298 cm^−1^ (*). (B) ‘CO_2_–N_2_’ ATR FTIR difference spectra in the presence of H_2_O (black) or D_2_O (magenta). The broad negative bands are assigned to ‘local’ water with *v*OH^−^ = 3000 cm^−1^ and *v*OH^−^ = 2280 cm^−1^. (C) Evolution of the bands assigned to HCO_3_^−^ (black) and *v*OH^−^ (blue) over time in the presence of 1–100% CO_2_ or 100% N_2_. Red lines represent linear fits of the first three data points after gas exchange to calculate the initial reaction velocity 
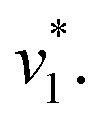
 (D) Plot of the apparent reaction velocity 
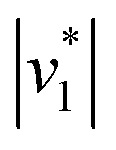
 of the initial HCO_3_^−^ formation (black) or initial water consumption (blue) in the CO_2_ hydration reaction.

Fig. 2A depicts a ‘CO_2_–N_2_’ FTIR difference spectrum recorded 25 s after addition of 10% CO_2_. The positive band at 2341 cm^−1^ corresponds to CO_2_ in solution.^13^ Further positive bands were observed at 1618 cm^−1^, 1358 cm^−1^, and 1298 cm^−1^, the latter as a shoulder. These bands are assigned to bicarbonate in solution,^14–16^*i.e.*, the asymmetric and symmetric stretching modes of CO_2_ (*v*_2_, *v*_3_) and the HCO_3_^−^ bending mode (*v*_4_). A broad negative band at 3000 cm^−1^ appeared in an energy regime corresponding to a strongly hydrogen-bonded network of ‘local’ water molecules,^21–23^ indicating that water is consumed during bicarbonate formation.

To confirm assignment of the observed bands, we investigated potential isotope effects. Adding ^13^CO_2_ gas instead of CO_2_ resulted in a specific down-shift of the CO_2_ band to 2278 cm^−1^ (*Δ*63), as well as the *v*_2_ and *v*_3_ bands of HCO_3_^−^ to 1586 cm^−1^ and 1322 cm^−1^ (*Δ*32 and *Δ*36, respectively). The isotope effect on the band at 1258 cm^−1^ was rather minor while the broad negative band at 3000 cm^−1^ was not affected by ^13^CO_2_, which is in line with our assignments of CO_2_/HCO_3_^−^ and ‘local’ water. We also investigated the influence of solvent isotope effects by exchanging the hydrated *Ks*Ccr protein film from H_2_O to D_2_O (Fig. 2B). In the presence of D_2_O, we observed a large down-shift of the negative band from 3000 cm^−1^ to 2280 cm^−1^ (*Δ*720) supporting our assignment of the water cluster. While the H/D exchange only had an insignificant effect on the *v*_2_ and *v*_3_ bands, the shoulder at 1298 cm^−1^ seemed to disappear in the deuterated sample. This is due to a ∼300 cm^−1^ down-shift that moves the signal out of the detection window of our FTIR setup and additionally confirms the COH (*v*_4_) assignment.^15^

Next, we studied the kinetics of the reaction between *Ks*Ccr and CO_2_. The difference spectra were simulated with contributions from CO_2_, H_2_O, and HCO_3_^−^ and corrected for unspecific changes (Fig. S3†). The resulting ‘peak area’ for each reactant was plotted against time. Fig. 2C shows the changes of HCO_3_^−^ (given by the sum of *v*_2_, *v*_3_, and *v*_4_) and ‘local’ water (*v*OH^−^) in *Ks*Ccr upon reaction with CO_2_. We titrated the enzyme in five consecutive steps changing the gas atmosphere to a continuous partial pressure of 1, 3, 10, 30, and 100% CO_2_ followed by exposure to 100% N_2_ after each CO_2_ step. Qualitatively, these data demonstrate that the intensity of the HCO_3_^−^ and water bands are proportional to the CO_2_ concentration in the atmosphere and that the CO_2_/HCO_3_^−^ conversion is reversible (eqn (1)). The initial velocity of CO_2_ hydration 
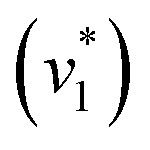
 was estimated by linear regression based on the first three data points after changing the atmosphere from N_2_ to CO_2_ for each step. We assume that the reaction velocity is not significantly affected by the back reaction due to the small build-up of HCO_3_^−^ within the first 15 s. A similar approach was chosen to quantify the initial velocity of HCO_3_^−^ dehydration 
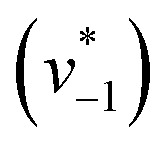
 initiated by removing CO_2_ from the gas atmosphere (Fig. S4†). The data yielded apparent reaction velocities 
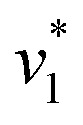
 and 
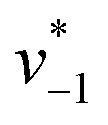
 that are specific for our experimental approach. Earlier, we explored how the humidity of concentrated protein films influences the velocity of substrate diffusion^24,25^ and its overall elastic properties.^26^ Now, we show that corresponding observations are made with *Ks*Ccr: when the humidity was reduced from 75% to 35% (determined *via* the OH stretching vibrations of H_2_O, see Fig. S3†) the velocity of CO_2_ hydration decreased accordingly (Fig. S5†). Although the spectroscopically measured velocities are lower than in solution assays^27^ our data facilitates a quantitatively significant comparison between samples under tightly controlled steady-state conditions. To demonstrate the catalytic activity of *Ks*Ccr in solution, we performed the ‘colorimetric’ analysis of CO_2_ hydration as pioneered by Wilbur and Anderson.^28^ Here, the injection of a defined amount of CO_2_-saturated buffer induces an acidification (eqn (1)), which leads to a bleach of a strong absorbance band of bromothymol blue that can be followed over time by UV/vis spectroscopy. Our data in Fig. S6† demonstrate that CO_2_ hydration in aqueous solution is much slower than in the presence of *Ks*Ccr or β-type carbonic anhydrase from *E. coli* (*Ec*CA) confirming the observed anhydrase activity of *Ks*Ccr.^29^

Fig. 2D shows how the calculated reaction velocities for HCO_3_^−^ formation and water consumption (given in absolute values 
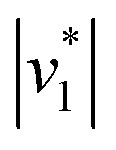
 to visually aid the comparison) depend linearly on the CO_2_ concentration. The experimental variation has been determined in repetitions of five (Fig. S7†). This confirms the proposed reaction model of pseudo-first order kinetics for enzymatic CO_2_ hydration in aqueous solution^30^ and highlights the quantitative connection between CO_2_, HCO_3_^−^, and water in the active site. Fig. S8† depicts a quantification of HCO_3_^−^ based on Na_2_CO_3_ reference samples and an analysis of the exponential correlation between CO_2_ partial pressure and HCO_3_^−^ concentration in the protein film. This facilitates the analysis of the initial velocity of the back reaction 
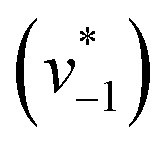
 as a function of bicarbonate concentration. The data in Fig. S4† suggest a higher reaction order and overall slower kinetics. We speculate that the apolar active site of *Ks*Ccr (Fig. 1) may slow down HCO_3_^−^ binding, which would impede the back reaction.

**Fig. 3:**
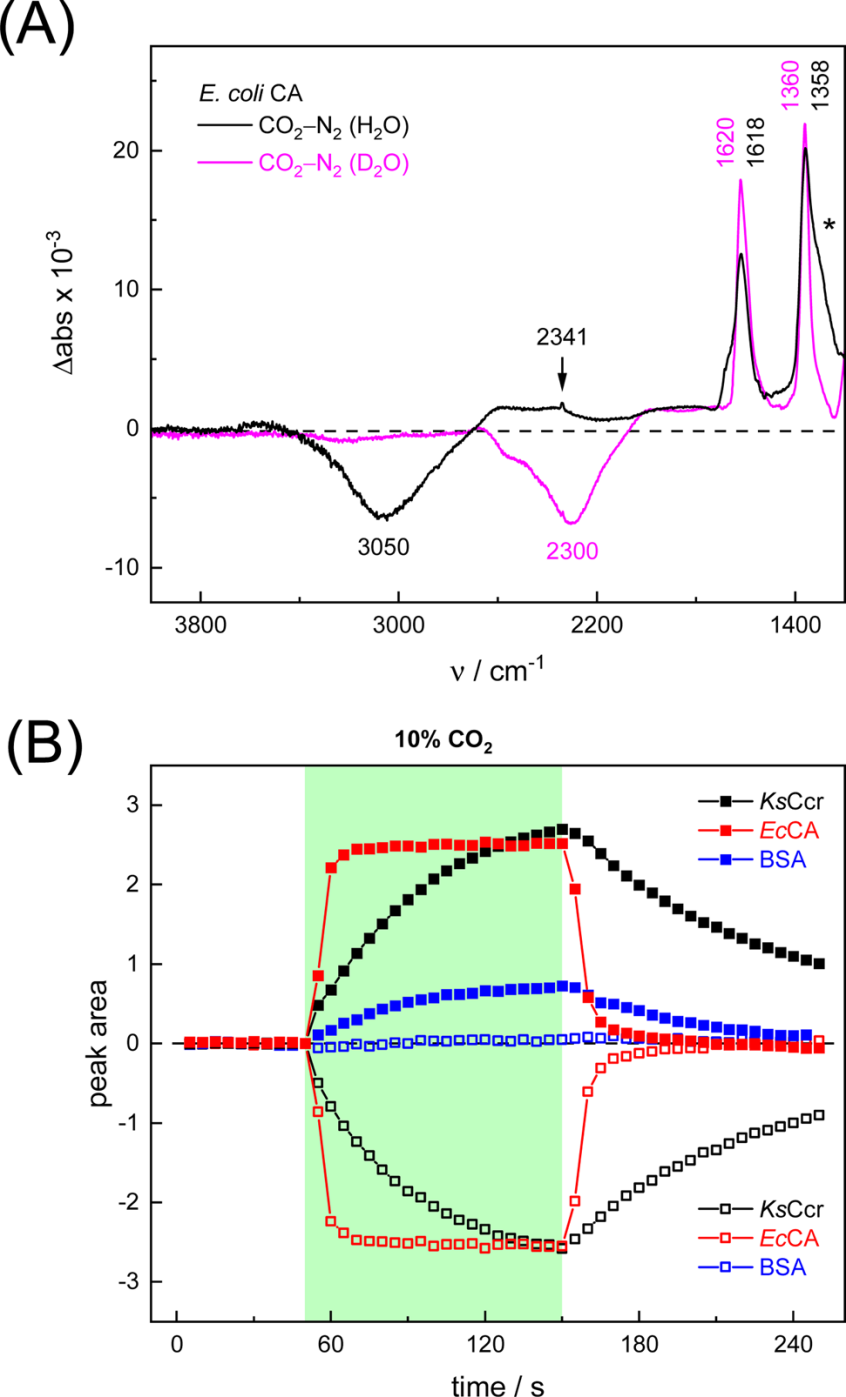
Infrared characterization of the reaction of *Ec*CA with CO_2_. (A) ‘CO_2_–N_2_’ ATR FTIR difference spectra in the presence of H_2_O (black) or D_2_O (magenta). These data show that *Ec*CA and *Ks*Ccr (Fig. 2) react very similar with CO_2_. (B) Plotting the evolution of spectral traces for HCO_3_^−^ (closed symbols) and *v*OH^−^ (open symbols) against time, the superior reaction velocity of *Ec*CA (red) over *Ks*Ccr (black) and BSA background (blue) becomes evident.

To verify and benchmark the CO_2_/HCO_3_^−^ conversion by *Ks*Ccr, we repeated the experiments with carbonic anhydrase *Ec*CA at conditions comparable to the experiments with *Ks*Ccr. The ‘CO_2_–N_2_’ FTIR spectrum for *Ec*CA after 25 s in the presence of 10% CO_2_ (Fig. 3A) is strikingly similar to the one observed for *Ks*Ccr (Fig. 2B) including the positive features for CO_2_ and HCO_3_^−^, as well as the negative water band at 3050 cm^−1^ (2300 cm^−1^ in D_2_O). However, Fig. 3B shows that *Ec*CA catalyses the CO_2_/HCO_3_^−^conversion nearly four times faster than *Ks*Ccr (
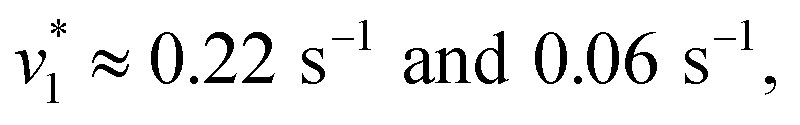
 respectively). The superior activity of *Ec*CA is observed in solution as well (Fig. S6†). For comparison unspecific CO_2_ conversion by bovine serum albumin (BSA, 
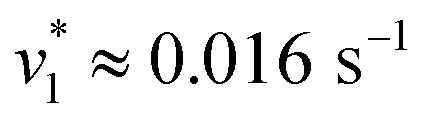
) is plotted in Fig. 3B. Note the lack of a negative band at 3000 cm^−1^

The CO_2_ conversion kinetics of *Ks*Ccr in Fig. 2 are in the range of uncatalyzed CO_2_ hydration in solution (Fig. S6†). Therefore, we probed background CO_2_ conversion in (i) water, (ii) buffer, and (iii) BSA as a generic biological crowder.^31^ No significant HCO_3_^−^ formation was observed with pure water, presumably due to the acidification in unbuffered solution (Fig. S2†). When recording ‘CO_2_–N_2_’ difference spectra on a drop of Tris/HCl buffer (2 μl, 25 mM, pH 8) approximately 10% HCO_3_^−^ formation was observed within the same time frame as in the *Ks*Ccr experiments (Fig. S2†) although much more CO_2_ is dissolved in the drop compared to *Ks*Ccr or *Ec*CA (Fig. S9†). As argued above, data based on liquid sample cannot be compared directly, therefore we formed a BSA protein film to probe uncatalyzed CO_2_ hydration under conditions comparable to the experiments with *Ks*Ccr or *Ec*CA. At pH 7–8, BSA shows up to 30% of the bicarbonate formation activity observed with *Ks*Ccr and a HCO_3_^−^/CO_2_ ratio similar to *Ks*Ccr or *Ec*CA. However, the unique water feature of *Ks*Ccr and *Ec*CA is shifted to 3320 cm^−1^, indicative of uncatalyzed CO_2_ hydration from bulk water (Fig. S9†). Accordingly, when the BSA solution was adjusted to pH values between 5–9, the observed HCO_3_^−^ formation resembles the pH profile of the CO_2_/HCO_3_^−^ couple in the absence of enzyme. Note that this is not the case for *Ks*Ccr: in the same pH range, this enzyme shows largely unchanged CO_2_ hydration activity (Fig. S9†). Overall, these controls demonstrated that *Ks*Ccr possesses a CA-like activity similar to the reaction of ‘true’ CAs.

### Substrate binding and hydrophilic residues influence anhydrase activity

**Fig. 4:**
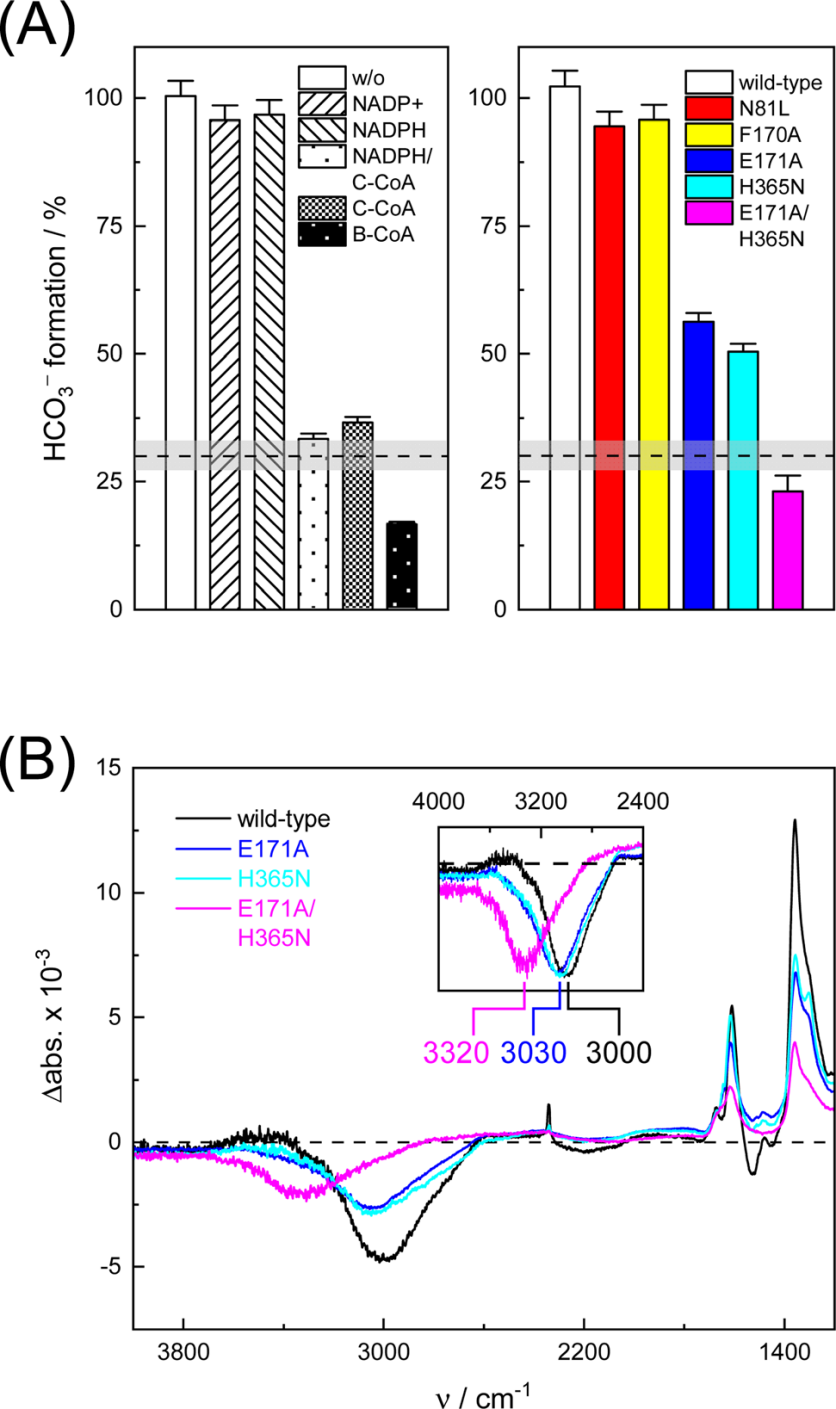
Bicarbonate formation activity of wild-type *Ks*Ccr and variants. (A) The HCO_3_^−^ peak area after 60 s in the presence of 10% CO_2_ is set to 100% for wild-type *Ks*Ccr and compared for different conditions (left panel) and active site variants (right panel) as annotated in the bar plot. About 30% CO_2_ hydration can be considered as unspecific background activity, which is indicated by the dashed line. (B) ‘CO_2_–N_2_’ FTIR difference spectra after 60 s highlight quantitative differences (*i.e.*, the intensity of the bicarbonate bands) and qualitative differences between wild-type *Ks*Ccr and variants: the inset shows an up-shift of the water band (scaled), most clearly visible for *Ks*Ccr double variant E171A/H365N (magenta).

In the next step, we investigated the CO_2_/HCO_3_^−^ conversion activity of *Ks*Ccr in the presence of NADPH, NADP^+^, native substrate crotonyl-coenzyme A (C-CoA), and side product butyryl-coenzyme A (B-CoA).^9–11^ We tested six different combinations: (i) *Ks*Ccr only, (ii) *Ks*Ccr + 10 mM NADP^+^, (iii) *Ks*Ccr + 10 mM NADPH, (iv) *Ks*Ccr + 10 mM NADPH + 1 mM C-CoA, (v) *Ks*Ccr + 1 mM C-CoA, and (vi) *Ks*Ccr + 1 mM B-CoA. Fig. 4A shows the HCO_3_^−^ peak area observed after 60 s in the FTIR difference experiments (*i.e.*, upon saturation of the signals, see Fig. S9†), normalized to wild-type *Ks*Ccr, which defines ‘100%’ bicarbonate formation. The experimental variation has been determined in repetitions of five (Fig. S7†). In these experiments, neither NADPH nor NADP^+^ affected bicarbonate formation, while the presence of C-CoA or B-CoA decreased band intensity down to 33% and 17%, respectively. Based on our reference experiments (Fig. S9†), we note that about 30% CO_2_ hydration can be considered as background activity, which is indicated by the dashed line in Fig. 4A. These data suggest that HCO_3_^−^ formation and carboxylation are mutually exclusive indicating that the CO_2_/HCO_3_^−^ conversion occurs only at the substrate-free active site of *Ks*Ccr. We speculate that the superior inhibition activity of B-CoA is related to the structural flexibility of this catalytic side product, which has been shown to fit the active site of *Ks*Ccr smoothly.^32^ These properties might affect unspecific binding site as well pushing the activity below the threshold.

We have shown previously that four amino acids play a key role in CO_2_ binding at the active site of *Ks*Ccr: histidine H365, glutamate E171, asparagine N81, and phenylalanine F170.^12^ H365 and E171 are involved in coordinating a conserved water molecule in bridging position (*μ*W), which is in hydrogen-bonding contact with water molecules that connect the active site with bulk water (Fig. 1). Asparagine N81 orients CO_2_ in the active site for the carboxylation reaction, and F170 shields the pocket from water.

To understand the molecular basis of CO_2_/HCO_3_^−^ conversion in *Ks*Ccr, we tested five active site variants. Qualitatively, the ‘CO_2_–N_2_’ FTIR difference spectra of single point mutants N81L, F170A, E171A, and H365N were similar to wild-type *Ks*Ccr. However, while N81L and F170A showed full conversion, bicarbonate formation of variants E171A and H365N was reduced by *ca.* 50% (Fig. 4A). Compared to H365N variant E171A showed slightly slower bicarbonate formation. In the *Ks*Ccr E171A/H365N double mutant both conversion and reaction velocity were found to be reduced even further (Fig. S10†). Fig. 4B highlights an interesting detail in the difference spectra of wild-type *Ks*Ccr, E171A, H365N, and E171A/H365N: the water band shifts from 3000 cm^−1^ to 3030 cm^−1^ in the single point mutants and all the way down to 3320 cm^−1^ in the double mutant. This indicates that *Ks*Ccr E171A/H365N has lost its CA-like activity and exhibits only unspecific CO_2_ hydration much like BSA that showed a similar ‘CO_2_–N_2_’ FTIR difference spectrum (Fig. S9†) and in agreement with the reduced CO_2_ hydration activity reported in Fig. 4A. In summary, these experiments suggest that the CA-like activity depends on the active site of *Ks*Ccr, and likely involves E171 and H365.

### Computer simulations reveal conformation-dependent CO_2_ binding and explain substrate inhibition

**Fig. 5:**
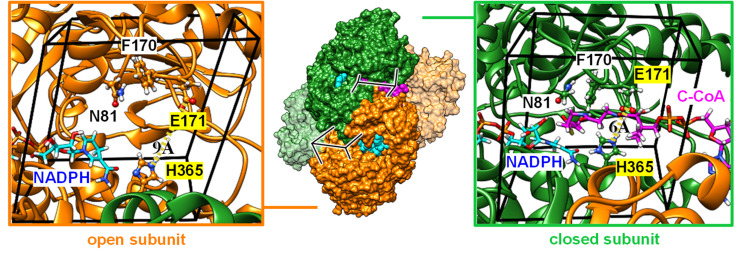
Computational model. Crystal structure of *Ks*Ccr (PDB ID 6NA4) in a dimer-of-dimers configuration with open subunits (orange, left panel) and closed subunits (green, right panel). A close-up to the active site for the open and closed subunit shows residues N81, F170, E171, and H365. Note that the E171/H365 distance shrinks from 9 Å to 6 Å in the closed subunit. A black box encloses the volume of the active site used to analyze the local CO_2_ concentration. NADPH is shown in cyan sticks, C-CoA is shown in magenta sticks. The later is exclusively found in the closed subunit (green, right panel).

To rationalize the observed inhibition of CA-like activity through substrate binding, we performed atomistic MD simulations in the presence of CO_2_ using the X-ray crystal structure of *Ks*Ccr that binds both NADPH and side product B-CoA (ternary complex, PDB ID 6NA4).^32^ Note that the enzyme is a tetramer that shows half-site reactivity, *i.e.*, exists as dimer of open and closed subunits (colored orange and green in Fig. 5). Compared to the open subunits the closed subunits contain the substrate and represent the catalytically active sites in the ternary complex. For our simulations, we replaced B-CoA by C-CoA and defined a specific volume box (Fig. 5) to calculate the CO_2_ binding free energy in the open and closed subunit. Notably, active site residues H365 and E171, which we associate with CA-like activity, adopted different geometries in the open and closed conformations. Compared to the open subunit, the distance between E171 and H365 was 3 Å shorter in the closed subunit and the conserved water molecule *μ*W was hydrogen-bonded between the two residues (Fig. 5). Additionally, we studied the X-ray structure without substrate (binary complex, PDB ID 6NA4) that presents similar geometries of both residues in the closed and open subunits.^32^

We presume that H365 can act as base in its neutral, monoprotonated state initiating proton abstraction from *μ*W and thus forming the hydroxyl ion for subsequent CO_2_ hydration. To determine the protonation state of H365 in different subunits of the binary and ternary complexes, we calculated the p*K*_a_ shift (Table S1†).^33^ For the open active site, the p*K*_a_ shift is negative (Δp*K*_a_ = −0.9 ± 0.1) indicating that H365 rather adopts a monoprotonated state. For the closed active site without substrate (binary complex), we also obtained a negative shift (Δp*K*_a_ = −0.6 ± 0.1) while the p*K*_a_ shift was positive in the presence of the substrate (Δp*K*_a_ = +1.2 ± 0.1), likely because of favorable interactions of the H365 with the negatively charged phosphate groups of C-CoA. Thus, H365 is monoprotonated in the empty, closed active site and capable of initiating proton abstraction from *μ*W. In the presence of substrate H365 most likely changes its protonation state, thus suppressing CA activity.

**Fig. 6:**
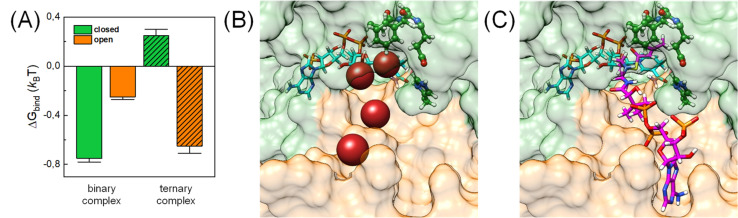
CO_2_ binding in the active site of *Ks*Ccr. (A) Binding free energy (*k*_B_*T*) of CO_2_ in the closed and open subunit of the binary complex with NADPH and the ternary complex with NADPH and C-CoA from MD simulations. (B) Most probable binding sites of CO_2_ in the closed active site of the binary complex (PDB ID 6NA6) represented as red spheres. (C) Representative snapshot of the ternary complex (PDB ID 6NA4), in which the substrate occupies the position of the CO_2_ binding sites. NADPH is shown in cyan, C-CoA is shown in magenta, and key residues are shown in green sticks.

In addition, CO_2_ binding to the active site plays an important role. To understand the influence of conformational changes and presence of the substrate on CO_2_, we carried out extensive MD simulations. From the ratio of local CO_2_ concentration in the active site volume (black box in Fig. 5) and the concentration in the bulk we calculated the CO_2_ binding free energy to the active site volume for the open and closed subunits of the binary and ternary complexes of *Ks*Ccr (
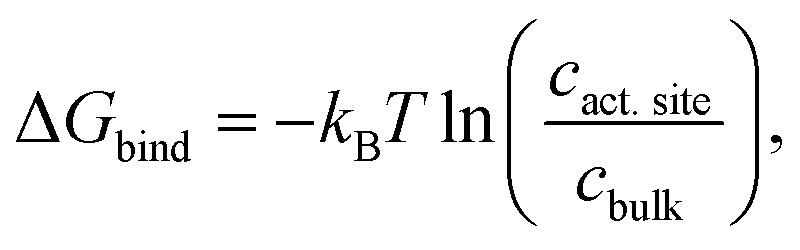
 see ESI†). Our calculations show that Δ*G*_bind_ of the closed subunit with substrate (ternary complex) is positive whereas the closed subunit in the binary complex without substrate presents the highest CO_2_ affinity which makes it more than two times more probable to find a CO_2_ molecule in the active site than in the bulk (Fig. 6A). The open subunit in the ternary complex also shows a significantly increased CO_2_ affinity but the distance between E171 and H365 is larger in the open active site, and no *μ*W molecule is observed suggesting a diminished catalytic activity. We then addressed the binding sites of CO_2_ in the closed active site without substrate where the affinity is highest. The binding sites connect active site interior and solvent, and the most buried ones are very close to H365, water molecule *μ*W, and E171 (Fig. 6B). Notably, the substrate in the ternary complex occupies the same positions as the CO_2_ binding sites (Fig. 6C).

In summary, our computer simulations show that the binary complex has a higher CO_2_ binding affinity compared to the ternary complex. The CO_2_ binding sites in the closed active site are next to H365 and the conserved water molecule *μ*W, so that the monoprotonated form of H365 will be able to abstract a proton from *μ*W to form the nucleophilic hydroxyl ion. The absence of NADPH or NADP^+^ is not expected to affect CO_2_ binding or H365 protonation because the coenzyme does not bind directly to the substrate binding site.^32^ In contrast, the presence of C-CoA or B-CoA in the ternary complex increases the p*K*_a_ of the putative proton acceptor H365 thereby eliminating its ability to activate *μ*W water by proton transfer, and simultaneously diminishes CO_2_ binding. This can explain the experimentally observed reduction of CA-like activity and is in line with the fact that the active enzyme ternary complex promotes CO_2_ fixation,^32^ and not CO_2_ hydration.

## Conclusions

In this study, we applied *in situ* ATR FTIR difference spectroscopy and computer simulations to investigate and understand the interaction of crotonyl-CoA carboxylase/reductase (*Ks*Ccr) with CO_2_. Our results show that *Ks*Ccr possesses a carbonic anhydrase-like activity, *i.e.*, the interconversion of CO_2_ and HCO_3_^−^ with water. This reaction is strongly suppressed in the presence of C-CoA, the natural substrate of *Ks*Ccr. Extensive MD simulations revealed how C-CoA suppresses CO_2_ binding and identified H365 as putative proton acceptor during CO_2_ hydration. Compared to wild-type *Ks*Ccr variant H365N indeed showed about 50% reduced anhydrase activity, similar to variant E171A. Our p*K*_a_ calculations rationalize how either H365 or E171 can serve as ‘base’ in the CO_2_ hydration reaction explaining the relatively large anhydrase activity of the single-residue variants. In contrast, only slow and unspecific CO_2_ hydration is observed with double variant E171A/H365N. In wild-type *Ks*Ccr, H365 and E171 form a hydrogen-bonding complex through an interstitial, bridging water molecule (*μ*W). The latter is in contact with a chain of water molecules that facilitate contact with bulk water. Upon CO_2_ hydration our FTIR data reveal the loss of a broad band at 3000 cm^−1^ (2280 cm^−1^ in D_2_O), which we assign to a strongly hydrogen-bonded water cluster, most likely including *μ*W.

Notably, we also observed very similar spectra for *Ec*CA, which we used as a reference to validate the experimental setup and confirm our interpretation of *Ks*Ccr's CA-like activity. *Ec*CA coordinates a zinc cofactor that catalyses the deprotonation of a bound water molecule to a hydroxide ligand (Zn^2+^–OH^−^) promoted by a nearby histidine base (Scheme 1). CO_2_ reacts with the ligand to HCO_3_^−^, which is clearly observed in our FTIR difference spectra as a positive contribution. In the following HCO_3_^−^ leaves the active site and is replaced by another water molecule. Binding of water ‘re-activates’ the cofactor, resulting in a broad negative band in our FTIR difference spectra, similar to what we have observed with *Ks*Ccr. Reported here for the first time, these results establish a unique spectral signature of CA activity, *i.e.*, the IR bands of bicarbonate and a strongly-hydrogen bonded cluster of ‘local’ water.

**Scheme 2:**
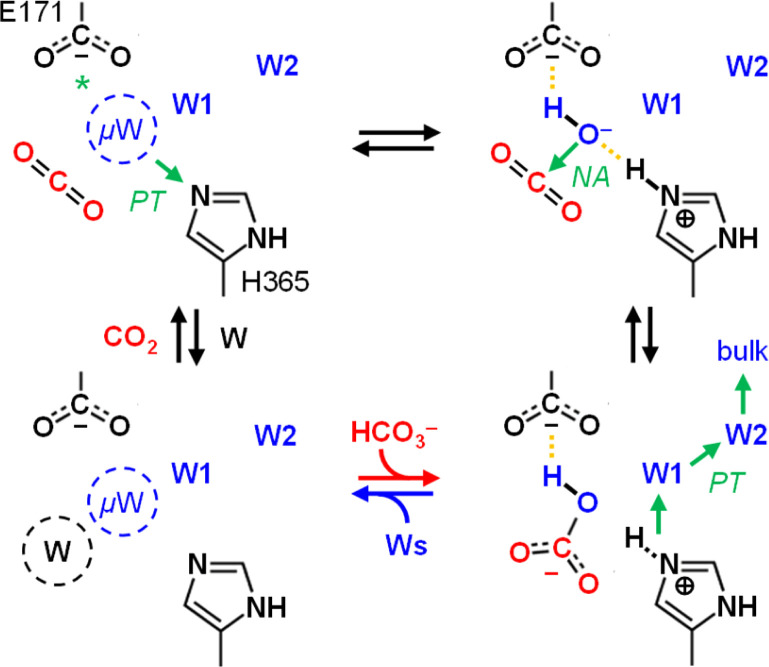
Proposed reaction mechanism. Top row, left to right: interstitial water *μ*W is deprotonated *via* H365 (*or E171 in the H365N variant) when the system adopts the closed state. The resulting *μ*OH^−^ species is stabilized by hydrogen bonds (dashed lines) and attacks a bound CO_2_ molecule to form bicarbonate HCO_3_^−^. Bottom row, right to left: when the system adopts the open state, HCO_3_^−^ leaves the active site and H365 releases a proton toward bulk solvent. Influx of water and CO_2_ primes *Ks*Ccr for another round of anhydrase activity.

The role and importance of a metal ion in CA has been discussed intensively.^8^ However, in 2021 Hirakawa *et al.* reported metal-free CAs in cyanobacteria and microalgae that appear to catalyse CO_2_ hydration in a purely organic environment.^34^ These observations are in line with our experiments on *Ks*Ccr that also suggest metal-independent CA-like activity. Based on our combined experimental and theoretical investigation of CO_2_ hydration in *K*sCcr, we propose a mechanism related to carbonic anhydrase (Scheme 2): (i) Once the enzyme adopts the closed state, *μ*W is deprotonated to a bridging hydroxide, *μ*OH^−^, with the neutral H365 residue serving as base (our p*K*_a_ calculations suggest that E171 may serve as base in H365 variants, see Table S1†). (ii) The carboxylate side chain of E171 accepts a hydrogen bond from *μ*OH^−^, which itself is stabilized *via* a hydrogen bond from the imidazole side chain of protonated H365. When CO_2_ is present in the active site *μ*OH^−^ will form HCO_3_^−^*via* a nucleophilic attack (NA, the reaction may involve additional water species, see Fig. S11†). (iii) Bicarbonate leaves the active site – potentially triggered by a transition from the closed to the open state – and deprotonation of H365 toward bulk solvent. We speculate that the protonated imidazolium cation is not stable in the absence of an interstitial water species. (iv) This transient opening of the hydrogen-bonding complex will allow intake of water and CO_2_ and prime the system for a new round of CO_2_ hydration.

Summing up, *in situ* ATR FTIR difference spectroscopy allowed investigating the interaction of different enzymes with CO_2_, providing a simple yet powerful approach to directly identify CA activity for a given biological sample. Moreover, our method is also suited to identify and characterize water clusters and will serve as an important tool to analyze CO_2_ hydration in biocatalysis,^35–38^ homogenous or heterogeneous catalysts,^39–41^ and (de-)hydration reactions in general.^42,43^

## Data availability

Data are available from the authors upon reasonable request.

## Author contributions

M. Tinzl, G. Stoffel, and H. Westedt produced and prepared the enzymes. A. Gomez performed the molecular dynamics simulations and p*K*_a_ calculations. S. T. Stripp designed and performed the spectroscopy experiments. H. Grubmüller, T. J. Erb, E. Vöhringer-Martinez and S. T. Stripp discussed the data. E. Vöhringer-Martinez designed the computer simulations. E. Vöhringer-Martinez and S. T. Stripp wrote the manuscript.

## Conflicts of interest

There are no conflicts to declare.

## Supplementary Material

SC-015-D3SC04208A-s001

## References

[cit1] Bierbaumer S., Nattermann M., Schulz L., Zschoche R., Erb T. J., Winkler C. K., Tinzl M., Glueck S. M. (2023). Chem. Rev..

[cit2] Wolfenden R. (2006). Chem. Rev..

[cit3] DiMario R. J., Machingura M. C., Waldrop G. L., Moroney J. V. (2018). Plant Sci..

[cit4] Christianson D. W., Fierke C. A. (1996). Acc. Chem. Res..

[cit5] Silverman D. N., McKenna R. (2007). Acc. Chem. Res..

[cit6] Krishnamurthy V. M., Kaufman G. K., Urbach A. R., Gitlin I., Gudiksen K. L., Weibel D. B., Whitesides G. M. (2008). Chem. Rev..

[cit7] Supuran C. T. (2016). Biochem. J..

[cit8] Kim J. K., Lee C., Lim S. W., Adhikari A., Andring J. T., McKenna R., Ghim C. M., Kim C. U. (2020). Nat. Commun..

[cit9] Erb T. J., Berg I. A., Brecht V., Müller M., Fuchs G., Alber B. E. (2007). Proc. Natl. Acad. Sci. U. S. A..

[cit10] Erb T. J., Brecht V., Fuchs G., Müller M., Alber B. E. (2009). Proc. Natl. Acad. Sci. U. S. A..

[cit11] Rosenthal R. G., Ebert M.-O., Kiefer P., Peter D. M., Vorholt J. A., Erb T. J. (2014). Nat. Chem. Biol..

[cit12] Stoffel G. M. M., Saez D. A., DeMirci H., Vögeli B., Rao Y., Zarzycki J., Yoshikuni Y., Wakatsuki S., Vöhringer-Martinez E., Erb T. J. (2019). Proc. Natl. Acad. Sci. U. S. A..

[cit13] Falk M., Miller A. G. (1992). Vib. Spectrosc..

[cit14] Davis A. R., Oliver B. G. (1972). J. Solution Chem..

[cit15] Rudolph W. W., Fischer D., Irmer G. (2006). Appl. Spectrosc..

[cit16] Garand E., Wende T., Goebbert D. J., Bergmann R., Meijer G., Neumark D. M., Asmis K. R. (2010). J. Am. Chem. Soc..

[cit17] Barth A. (2007). Biochim. Biophys. Acta, Bioenerg..

[cit18] Lórenz-Fonfria V. A. (2020). Chem. Rev..

[cit19] Stripp S. T. (2021). ACS Catal..

[cit20] Fahrenfort J. (1961). Spectrochim. Acta.

[cit21] Keutsch F. N., Saykally R. J. (2001). Proc. Natl. Acad. Sci. U. S. A..

[cit22] Scatena L. F., Brown M. G., Richmond G. L. (2001). Science.

[cit23] Wang H., Wagner J. C., Chen W., Wang C., Xiong W. (2020). Proc. Natl. Acad. Sci. U. S. A..

[cit24] Duan J., Mebs S., Laun K., Wittkamp F., Heberle J., Happe T., Hofmann E., Apfel U.-P., Winkler M., Senger M., Haumann M., Stripp S. T. (2019). ACS Catal..

[cit25] Senger M., Mebs S., Duan J., Shulenina O., Laun K., Kertess L., Wittkamp F., Apfel U.-P., Happe T., Winkler M., Haumann M., Stripp S. T. (2018). Phys. Chem. Chem. Phys..

[cit26] Yakimets I., Paes S. S., Wellner N., Smith A. C., Wilson R. H., Mitchell J. R. (2007). Biomacromolecules.

[cit27] Ho C., Sturtevant J. M. (1963). J. Biol. Chem..

[cit28] Wilbur K. M., Anderson N. G. (1948). J. Biol. Chem..

[cit29] Smith K. S., Smith F. G. (2000). FEMS Microbiol. Rev..

[cit30] Kern D. M. (1960). J. Chem. Educ..

[cit31] Löwe M., Kalacheva M., Boersma A. J., Kedrov A. (2020). FEBS J..

[cit32] DeMirci H., Rao Y., Stoffel G. M., Vögeli B., Schell K., Gomez A., Batyuk A., Gati C., Sierra R. G., Hunter M. S., Dao E. H., Ciftci H. I., Hayes B., Poitevin F., Li P.-N., Kaur M., Tono K., Saez D. A., Deutsch S., Yoshikuni Y., Grubmüller H., Erb T. J., Vöhringer-Martinez E., Wakatsuki S. (2022). ACS Cent. Sci..

[cit33] Thurlkill R. L., Grimsley G. R., Scholtz J. M., Pace C. N. (2006). Protein Sci..

[cit34] Hirakawa Y., Senda M., Fukuda K., Yu H. Y., Ishida M., Taira M., Kinbara K., Senda T. (2021). BMC Biol..

[cit35] Zastrow M. L., Peacock A. F. A., Stuckey J. A., Pecoraro V. L. (2012). Nat. Chem..

[cit36] Rettberg L. A., Stiebritz M. T., Kang W., Lee C. C., Ribbe M. W., Hu Y. (2019). Chem.–Eur. J..

[cit37] Meneghello M., Oliveira A. R., Jacq-Bailly A., Pereira I. A. C., Leger C., Fourmond V. (2021). Angew. Chem., Int. Ed..

[cit38] Shevela D., Do H.-N., Fantuzzi A., Rutherford A. W., Messinger J. (2020). Biochemistry.

[cit39] Parkin G. (2004). Chem. Rev..

[cit40] Koziol L., Valdez C. A., Baker S. E., Lau E. Y., Floyd W. C., Wong S. E., Satcher J. H., Lightstone F. C., Aines R. D. (2012). Inorg. Chem..

[cit41] Cobb S. J., Badiani V. M., Dharani A. M., Wagner A., Zacarias S., Oliveira A. R., Pereira I. A. C., Reisner E. (2022). Nat. Chem..

[cit42] Kobayashi S., Manabe K. (2002). Acc. Chem. Res..

[cit43] Li G., Wang B., Resasco D. E. (2020). ACS Catal..

