# Supplementary material for: Infrared spectroscopy reveals metal-independent carbonic anhydrase activity in crotonyl-CoA carboxylase/reductase
Source: Chem Sci. 2024 Feb 29;15(13):4960–8. doi: 10.1039/d3sc04208a (PMC10966969; doi:10.1039/d3sc04208a)
Supplement: SC-015-D3SC04208A-s001 [file SC-015-D3SC04208A-s001.pdf]

Electronic Supplementary Material (ESI) for Chemical Science.
This journal is © The Royal Society of Chemistry 2024

Supporting Information for

Infrared Spectroscopy Reveals Metal-independent Carbonic Anhydrase Activity in Crotonyl-CoA Carboxylase/Reductase

Aharon Gomez[1†], Matthias Tinzl[2†], Gabriele Stoffel[2], Hendrik Westedt[2], Helmut Grubmüller[3], Tobias J. Erb[2,4], Esteban Vöhringer-Martinez[1*], Sven T. Stripp[5,6*]

Email: evohringer@udec.cl, s.stripp@tu-berlin.de

Table of contents

1.) Experimental Methods

2.) Computational Methods

3.) Figures S1–S11

4.) References

Experimental Methods

Protein production and purification. Wild-type crotonyl-CoA carboxylase/reductase from *Kitasatosporae setae* (*Ks*Ccr) and amino acid variants were produced and purified as previously described[1]. Briefly, His-tagged protein was expressed in *E. coli* BL21 (DE3) or *E. coli* BL21 (AI) from a pET16b vector. Freshly transformed cells were grown in TB medium containing ampicillin (100 µg/ml) at 37°C to an OD_{600} of around 0.6. The expression was initiated by addition of 1 mM IPTG (isopropyl-D-β-thiogalactopyranoside) for BL21 (DE3) or 1 mM IPTG and 0.025% (w/v) L-arabinose for BL21 (AI), before the temperature was reduced to 25°C. After 12–18 h cells were harvested by centrifugation at 10 000 g for 15 min. Cell pellets were resuspended in wash buffer (50 mM Tris, 500 mM NaCl, 1M L-proline, 20 mM imidazole, pH 7.5) and lysed by sonication or microfluidizing. Cell lysate was clarified by centrifugation at 5000 g for 15 min and loaded onto a Ni-NTA column. After washing with 3–4 column volumes of wash buffer, protein was eluted using 5–10 ml elution buffer (50 mM Tris/HCl, 500 mM NaCl, 1M L-proline, 250 mM imidazole, pH 7.5). After elution, samples were pooled and the buffer was exchanged to 25 mM Tris/HCl pH 8 or a 'mixed buffer' solution including 25 mM MES, Tris, and PIPPS at pH 5–9. Protein concentrations were determined by Uv/vis measurements using an extinction coefficient at 280 nm of ε_{280}= 66.4 mM^{-1} cm^{-1}. Proteins were frozen and stored at -20°C or -70°C.

His-tagged β-carbonic anhydrase from *E. coli* was expressed in *E. coli* BL21 (DE3) from the expression vector in the ASKA collection[2] (available in house). Freshly transformed cells were grown in TB medium containing ampicillin (100 µg/ml) at 37°C to an OD_{600} of 0.8–1. Expression was initiated by addition of 500 µM IPTG and the temperature was reduced to 23°C. After 16 h cells were harvested by centrifugation at 7500 g for 15 min at 4°C. Cell pellets were resuspended in 'wash buffer' (50 mM Tris/HCl, 500 mM NaCl, pH 7.5) and treated with 10 mg/mL DNAse I and 5 mM $MgCl_2$ on ice for 20 min upon followed by lysis with sonication. The cell lysate was clarified by centrifugation at 45 000 g for 45 min at 4°C and loaded onto a Ni-NTA column. After washing with 20 column volumes of wash buffer, the protein was eluted using elution buffer (50 mM Tris/HCl, 500 mM NaCl, 500 mM imidazole, pH 7.5). After elution, samples were pooled and the buffer was exchanged to 25 mM Tris/HCl pH 8. The protein concentration was determined by Uv/vis and then frozen in N_2(l) and stored at -20°C.

Thioester Synthesis and Purification. Crotonyl- and butyryl-CoA esters were synthesized using the anhydride method as previously described.[4] Briefly, 200 mg coenzyme A (CoA, 0.25 mmol, 1 eq.) were dissolved in 5 mL 0.5 M $NaHCO_3$ and the solution was cooled on ice. 1.6

eq. of the corresponding anhydride (crotonic anhydride: 65 µl, 0.4 mmol butyric anhydride: 65 µl, 0.4 mmol) was added and the solution was stirred on ice for 45 min and then directly injected into the HPLC-MS for purification.

Ethylmalonyl-CoA was synthesized by chemical coupling of crotonic anhydride with CoA followed by the addition of an ECR variant of *C. crescentus* (*Ccr*C_{PAG}), NADPH, and $KHCO_3$ as described previously.[5] 10 mg (0.0125 mmol, 1 eq.) CoA was dissolved in 1 mL 0.5 M $NaHCO_3$.The mixture was cooled on ice and crotonic anhydride (3.2 µl, 0.0081 mmol, 1.6 eq) was added. After completion (around 30 min, the reaction mixture was added to 8 mL of 250 mM Tris/HCl pH 7.5 containing 30 mM NADPH, 250 mM $KHCO_3$, and 100 nM *Ccr*Cc IA/CP/FG. The reaction was incubated for 30 min at 30°C, quenched with final a concentration of 5% formic acid and directly used for HPLC purification.

All synthesized CoA-thioesters were purified using a 1260 Infinity LC system (Agilent) using a Gemini 10 mm NX-C18 110 Å, 100 x 21.2 mm, AXOA packed column (Phenomenex) as previously described.[5] Method: flow rate: 25 ml/min starting with 5 min of 5% MeOH in 50 mM ammonium bicarbonate pH 8.2, gradient 5–40 % MeOH in 15 min, 2 min wash at 95% MeOH, a re-equilibration 3 min at 5% MeOH. Purified CoA-esters were lyophilized and concentrations were determined by Uv/vis absorbance using extinction coefficients of ε_{260}= 16.4 mM^{-1} cm^{-1} for saturated CoA esters and ε_{260}=22.4 mM^{-1} cm^{-1} for unsaturated CoA esters.[4]

Carbonic anhydrase assay. In solution, CO_2 hydration activity was probed by UV/vis spectroscopy according to established protocols.[6] All experiments were performed in the CO_2-free atmosphere of a N_2-filled glovebox (Coy Lab), exploiting an USB spectrometer (Ocean Optics Optics USB2000) with a halogen/deuterium light source (Ocean Optics DH-2000). The cuvette holder was set to a constant temperature of 4°C *via* a circulation pump (Julabo F20/HC) operating outside the glovebox. Ahead of the experiment, 400 µl Tris/HCl buffer (25 mM, pH 8) was mixed with 100 µl bromothymol blue solution (10 mg/l) and 100 µl dilute protein solution (10 mg/l *Ec*Ca or *Ks*Ccr) or 100 µl buffer. All samples were store on ice. A glass vial containing 20 mL ice-cold buffer was purged with CO_2 for 2 h, sealed with a rubber stopper afterwards, and transferred into the glovebox. For the experiment, 600 µl sample was injected into a 1 mL quartz cuvette (d = 1 cm). Electronic spectra were recorded with a temporal resolution of 0.9 s. Approximately 60 s after the acquisition of spectra was started, 400 µl carbonated buffer was injected into the cuvette for a total volume of 1 mL. The cuvette was capped directly afterwards. The decrease of the characteristic bromothylmol blue band at 618 nm could then be followed over time to quantify acidification of the medium as a result of CO_2

hydration. The solution dropped from pH 8 to pH 6.2 over the course of the experiment. For the analysis of data, all spectra were fitted with a single Lorentz function (peak position = 618 nm, fwhm = 83 nm) and a polynomial background accounting for stray a light and baseline shifts. The "peak area" was then plotted as a function of time.

Infrared spectroscopy. The FTIR spectrometer (Tensor27, Bruker Optik, Germany) was equipped with a triple-reflection ZnSe/Si crystal ATR cell (Smith Detection, USA) and placed in an anaerobic chamber (Coy Laboratories, USA). The atmosphere was adjusted to 99% N_2 and 1% H_2 with no O_2, CO_2, or H_2O. Infrared spectra were recorded with 80 kHz scanning velocity at a spectral resolution of 2 cm^{-1}. Under these conditions, the time-resolution of data acquisition is in the range of 1 s (one interferometer scan in the forward/backward direction).

To prepare a protein film, 1 µL protein solution (100–200 µM) was pipetted onto the silicon crystal of the ATR cell and enclosed by a custom-made gas titration cell to concentrate the sample under a stream of dry N_2 'carrier' gas (1 L/min), as reported earlier.[7] Once a partially de-hydrated protein film was formed, the gas was sent through a reservoir of 150 mL aqueous buffer (H_2O or D_2O), creating an aerosol that was used to re-hydrate the hygroscopic protein film. The reaction with CO_2 was started by adding 1–100% CO_2 to the carrier gas while recording spectra with a time resolution of 20 s. The reaction with CO_2 isotopes was probed by pressuring the closed gas cell with 1 bar $^{13}CO_2$.

Computational Methods

Alchemical Free Energy Calculations and Molecular Dynamics Simulations. The computational models were based on the crystal structure of the ternary complex of *Ks*Ccr (PDB ID 6NA4). This corresponds to a tetrameric structure that adopts a pair of dimers geometry. Each dimer is comprised by a closed and an open subunit (subunits A/C and B/D), where the closed state is the catalytically competent active site conformation. Each subunit contains the cofactor NADPH and the two closed subunits bear the substrate analogue butyryl-CoA (subunits A and B). The latter was modified to restore the original substrate crotonyl-CoA (*i.e.*, one hydrogen atom was removed from the β-carbon and the α-carbon).

To establish if H365 could act as a base in bicarbonate formation we performed alchemical free energy calculations to determine its pK_a shift compared to a histidine residue in a fully solvated peptide with methylated carboxy and amino terminals. The histidine peptide was added to the simulation box containing the protein, cofactor, substrate, and solvent. We applied alchemical free energy calculations to obtain the change in free energy $\Delta\Delta G^{o}$, when a proton is transferred

from the H365 residue in the open or closed active site to the fully solvated histidine residue. The pK_a shift of H365 in the open and closed active site results from:

$$\Delta pK_a = \frac{\Delta\Delta G^{o}}{2.303RT}$$

Topologies for the protonated and deprotonated states of the two residues were created with the PMX toolset[8,9] in GROMACS 2019.3[10] software package using the CHARMM36m[11] force field and the CHARMM TIP3P water model[12] in combination with the CHARMM general Force Field CGenFF[13,14] to describe CO_2 substrate and cofactor. Specific parameters for NADPH and the CoA fragment of crotonyl-CoA were taken from Pavelites *et al.*[15] and Aleksandrov *et al.*[16], respectively.

Starting with the protonated H365 either in the open or closed active site, we placed the protein tetramer in a dodecahedric water box using gmx solvate, such that the smallest distance between any atom of the protein and box boundary was larger than 1.5 nm. A methylated peptide NME-His-ACE was placed at least 3.5 nm away from H365 replacing water molecules. Potassium and chloride ions were added to neutralize the system, reaching a physiological concentration of 125 mmol/L. Energy minimization was performed using the steepest descent algorithm for 50000 steps for each system followed by a 1 ns equilibration applying position restraints on the protein backbone, peptide backbone and ligands in the NVT ensemble, and subsequently for 5 ns in the NPT ensemble at 1 atm pressure and a temperature of 298K using an integration time step of 2 fs. The velocity rescaling thermostat[17] and Berendsen[18] pressure coupling were used with coupling coefficients of τ = 0.1 ps and τ = 2 ps, respectively. All bond lengths of the protein, peptide and ligands were constrained using LINCS with an expansion order of 4. Electrostatic interactions were calculated using Particle-Mesh-Ewald[19], with a real space cutoff of 12 Å and a Fourier spacing of 1.6 Å. For the van-der-Waals interactions a cutoff of 12 Å and a switching function starting at 10 Å was used. Equilibrium free energy calculations were performed for 5 ns per lambda value with 11 [0.00, 0.10, 0.20, 0.30, 0.40, 0.50, 0.60, 0.70, 0.80, 0.90, 1.00] values for the coulomb interactions, 9 [0.00, 0.13, 0.25, 0.38, 0.50, 0.63, 0.75, 0.88, 1.00] for the van der Waals interactions, and 7 [0.00, 0.15, 0.30, 0.50, 0.70, 0.85, 1.00] for the bonded interactions in a NVT ensemble with positions restraints over the protein and peptide backbone and ligands at 298K with stochastic dynamics. The free energy differences (**Tab. S1**) were estimated by Bennet Acceptance Ratio (BAR)[20] included in GROMACS as gmx bar.

Table S1. Free energy difference ($\Delta\Delta G°$) between the protonated H365 of wild-type *Ks*Ccr in the open or closed active site and E171 in *Ks*Ccr H365N and the model peptide with unprotonated histidine of glutamate in aqueous solution. Error estimation results from BAR free energy estimation. The pK_a shift of H365 and E171 was calculated according to the equation above.

	**ΔΔG° [kJ/mol]**	**Δ*p*K$_a$**
**H365**<br>open active site<br>wild-type	-5.4 ± 0.2	-0.9 ± 0.1
**H365**<br>closed active site<br>wild-type<br>(binary complex)	-3.7 ± 0.5	-0.6 ± 0.1
**H365**<br>closed active site<br>wild-type w/ C-CoA<br>(ternary complex)	7.0 ± 0.5	+1.2 ± 0.1
**E171**<br>closed active site<br>H365N variant	11.92 ± 1.5	+2.1 ± 0.3

Our results suggest that H365 is monoprotonated (neutral) in absence of substrate for the open or closed states of the active site under the experimental condition of pH=8. Both conformations showed a negative pK_a shift, related to a more stable configuration for the monoprotonated state in the active site compared to the fully solvated peptide in solution. For the closed state with C-CoA, a positive pK_a shift indicates H365 being doubly protonated (charged) compared to the fully solvated peptide in solution.

Based on the most probable protonation state of H365 in the closed or open active site we build new systems to analyze the local concentration of CO_2 in the active sites. We started from the reported crystal structure for *Ks*Ccr (PDB ID: 6NA4) as previously mentioned and we generated two different configurations. The NADPH only complex with all four H365 monoprotonated and the NADPH/C-CoA ternary complex, which has one Crotonyl-CoA molecule and the H365 doubly protonated in each of the closed active sites. Hydrogen addition and topology generation for the systems was done in GROMACS 2019.3 software package[10]. Protonation states of titratable residues were assigned using Propka 3.1[21]. The interactions in the molecular system

were described with the CHARMM36m[11] force field and the CHARMM TIP3P water model[12] in combination with the CHARMM general Force Field CGenFF[13,14] to describe CO_2, substrate and cofactor. Parameters for CO_2 were validated with hexane/water partition coefficients and free energy calculations. Specific parameters for NADPH and the CoA fragment of crotonyl-CoA were taken from Pavelites *et al.*[15] and Aleksandrov *et al.*[16], respectively. The protein system was embedded in a rectangular box of water molecules leaving a 10 Å buffer region between the protein and box edges. Potassium and chloride ions were added to neutralize the system, reaching a physiological concentration of 125 mmol/L. Energy minimization was performed using the steepest descent algorithm for 50000 steps for each system followed by a 1 ns equilibration applying position restraints on the protein backbone, peptide backbone and ligands in the NVT ensemble, and subsequently for 5 ns in the NPT ensemble at 1 atm pressure and a temperature of 298K using an integration time step of 2 fs. The velocity rescaling thermostat[17] and Berendsen[18] pressure coupling were used with coupling coefficients of τ = 0.1 ps and = 2 ps, respectively. All bond lengths of the protein, and ligands were constrained using LINCS with an expansion order of 4. Electrostatic interactions were calculated using Particle-Mesh-Ewald[19], with a real space cutoff of 12 Å and a Fourier spacing of 1.6 Å. For the van-der-Waals interactions a cutoff of 12 Å and a switching function starting at 10 Å was used.

After the initial equilibration, 100 CO_2 molecules were added replacing water molecule. To enhance the sampling the simulations presented ~60 times the experimental concentration[1] of CO_2 (c= 55mM). Energy minimization was performed using the steepest descent algorithm for 50000 steps for each system followed by a 1 ns equilibration applying position restraints on the protein backbone, peptide backbone and ligands in the NVT ensemble, and subsequently for 5 ns in the NPT ensemble at 1 atm pressure and a temperature of 298K using an integration time step of 2 fs. After the pressure is equilibrated, production dynamics in the NVT ensemble were performed keeping position restraints on the protein alpha carbons and NADPH heavy atoms to keep the protein conformation fixed. In the NADPH system the distance between R276 and NADPH's adenine moiety was restraint to its initial value. For the NADPH/C-CoA, distance between residue R99 and crotonyl-CoA's phosphate group, R303 and C-CoA's adenine moiety, the reactive Crotonyl-CoA's C_{β} with the NADPH's C_4 and H409 with the NADPH's phosphate group for the closed subunits were restraint to its initial value to explore CO_2 distributions in the defined states of the catalytic cycle of the enzyme. Multiples replicas were performed generating a total of 20 µs of simulation time for each system, the coordinates were saved every 100ps resulting in $2x10^5$ configurations available for analysis.

CO_2 concentration and binding sites in the active site. To analyze carbon dioxide distribution and binding sites around the protein and in its active sites, we use the gromaρs [22] analysis tools: The position of the atoms is expanded by 3-D Gaussians to represent atomic densities (widths of the gaussians equal experimentally determined atomic scattering factors). This procedure is carried around at every frame of the trajectory on a grid with a resolution of 0.1 nm and time averaged over trajectories.

We want to compare the CO_2 concentration in the active site in the closed and open state and the absence or presence of substrate compared to the bulk concentration. We start defining the bulk concentration as the solution concentration ($C_{solution}$). This concentration corresponds to the number of carbon dioxide molecules inside the simulation box divided by the Solvent Accessible Volume (SAV). The SAV is a magnitude that accounts for the points in space that carbon dioxide molecules can access and represents the volume of the simulation box excluding the volume of the Protein, NADPH and C-CoA atomic densities. SAV is modulated by the movement of all atoms in the molecular dynamics simulation. To account for this effect, we calculated the time averaged $\langle SAV \rangle$ to estimate the solution concentration.

$$C_{solution} = \frac{N_{CO_2}}{\langle SAV \rangle}$$

To estimate the SAV we expand the heavy atoms positions for the protein, NADPH and C-CoA as atomic densities for all frames at 100 ns time intervals. This density is time averaged and accounts for different conformations of protein's side chains. Once the density map is obtained, we set a threshold isosurface value of 0.1 [a.u], which recovers the overall shape of the residue side chains, cofactors and substrates in the protein complex. Finally, to get the SAV we count all grid points (g_i) with a density lower than the threshold, which corresponds to the volume not occupied for the protein, cofactor and substrate atoms, thus accessible for the solvent and CO_2.

$$SAV = \sum_{i}^{N} g_i ; \forall \rho(g_i) < threshold$$

With the CO_2 concentration in the bulk/solution we focused on the local concentration in the active site. We define the active site as the volume inside a box containing all key residues for the reaction (black box **Figure 5A**), the nicotinamide ring and the crotonyl-CoA fragment until the pyrophosphate. To obtain the local concentrations at the active sites, the number of $CO_{2,AS}$ molecules and the $\langle SAV \rangle_{AS}$ inside the boxes must be calculated. The number of carbon dioxide

molecules can be calculated from the atomic density inside the active site box. For the active site $\langle SAV\rangle_{AS}$ we followed the same procedure described for the whole protein but considering only the volume of the active site box.

To estimate the number of CO_2 molecules we start from the density map obtained by gromaρs, this is a 3D discretization of space with density values for CO_2 at every grid point, then we space average all densities for the active site, summing up all the grid points and dividing them by the value of one CO_2 molecule. This quantity gives us the number of CO_2 molecules inside the active site, which is then divided by the active site's SAV to get concentration values:

$$c_{Active\ site} = \frac{N_{CO_{2,AS}}}{\langle SAV\rangle_{AS}}$$

This concentration is derived as the time average of different configurations of CO_2 molecules inside the active site. This volume is corrected accounting only for the accessible volume of the solvent, which is common to the CO_2 molecules. With both active site concentrations and the solution concentration, we can calculate the enrichment in the active site values from which the binding free energy is calculated as described in the manuscript:

$$\frac{c_{ac.site}}{c_{solution}}$$

To define binding sites, we use the CO_2 density maps obtained from the analysis with gromaρs in the active sites. We first identified intervals of 100 ns where the average CO_2 concentration in the active is at least twice the solvent concentration to select configurations where carbon dioxide is bound in the active site. Binding sites were defined using the volumes enclosed by isosurfaces of CO_2 concentration selecting an isosurface value 20 times larger than the bulk concentration (see **Figure S10** below). The centers of the volumes define the center of the binding sites shown in **Figure 6** in the main script.

Figure S1–S11

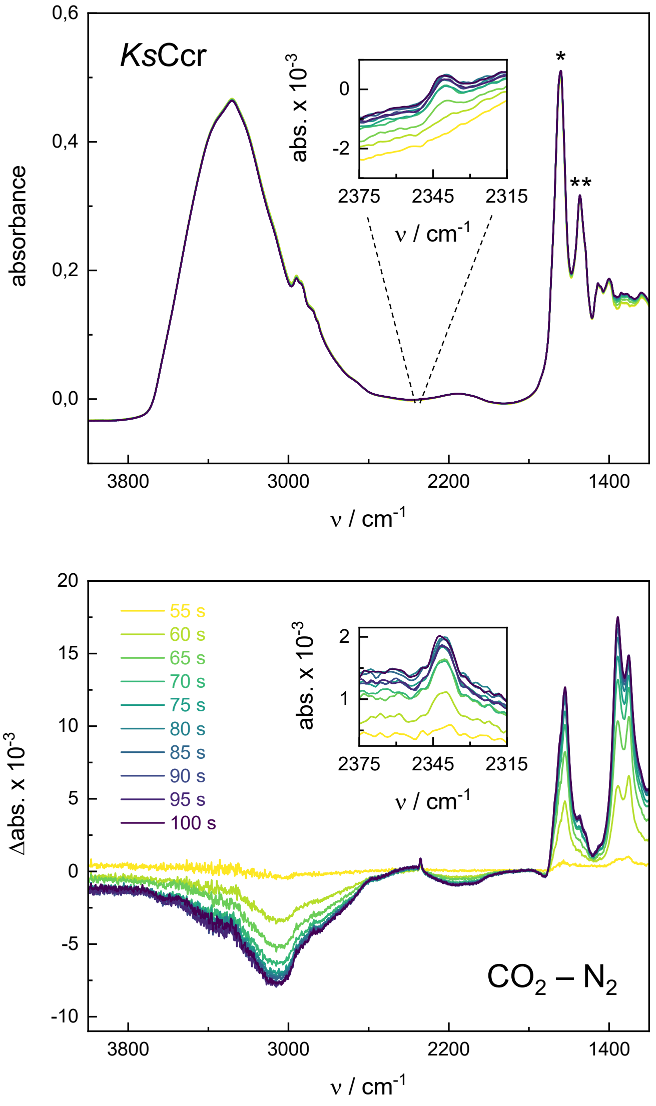

Figure S1. Upper panel: Time series (50–100s) of FTIR absorbance spectra of *Ks*Ccr reacting with 10% CO_2. The inset shows how CO_2 non-specifically accumulates in the well-hydrated film (a single band at 2341 cm^{-1}). The bands of the protein backbone are marked (* Amide I and ** Amide II). Lower panel: Time series of 'CO_2 – N_2' FTIR difference spectra, calculated by subtracting the last spectrum under 100% N_2 (t_0=50 s) from all spectra under 10% CO_2 (55–100 s). The data illustrate the increase and decrease of specific and unspecific features over time (see **Figure S3** for a more detailed description). Specific negative features are assigned to the 'background' (100% N_2), positive features are assigned to the 'signal state' accumulating under 10% CO_2. The inset shows how CO_2 accumulates in the film.

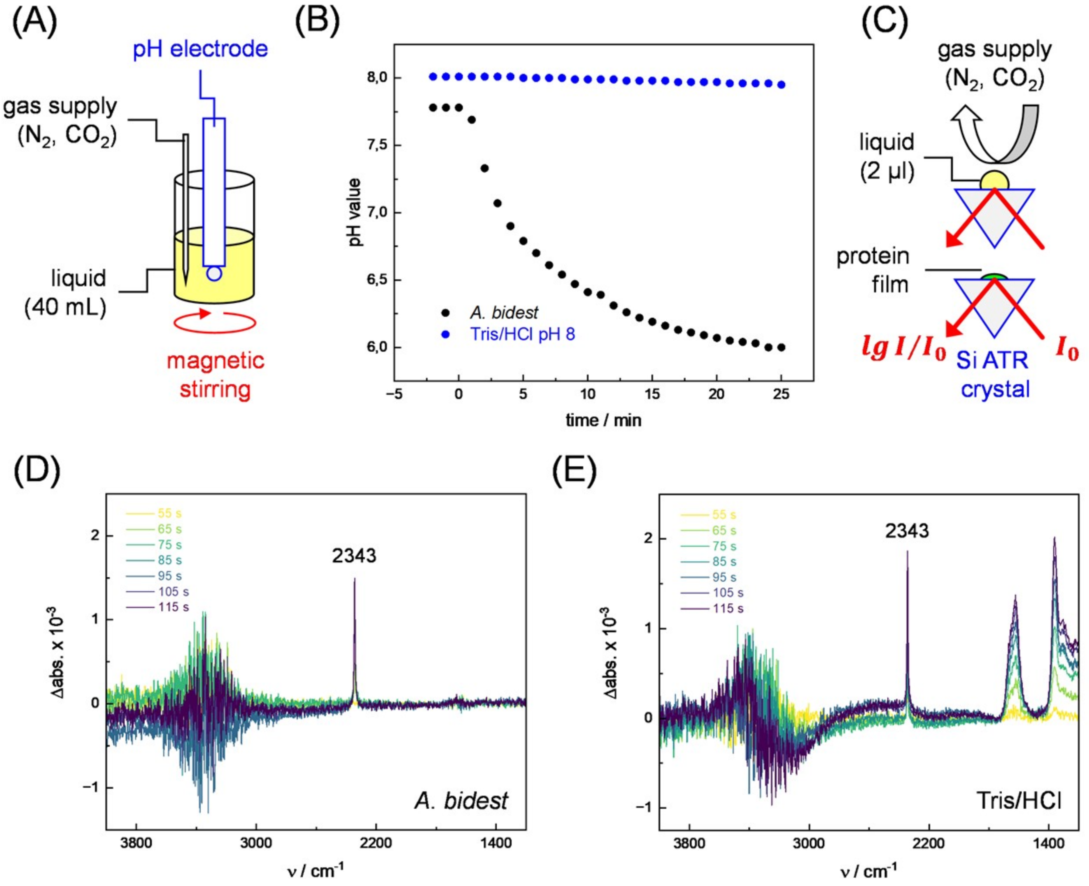

Figure S2. (A) Directly measuring the pH of the sample film in the ATR FTIR experiments is impossible. To probe the stability of pH in the presence of 10% CO_2 the following representative experiment was attempted: We purged 40 mL *A. bidest* or buffer solution (200 25 mM Tris/HCl pH 8) with 100 mL/min N_2 for 15 minutes at 22°C and a stirring speed of 300 rpm). This removed atmospheric CO_2 from the solution. Then, the experiment was started by measuring the pH with a tabletop pH electrode (Thermo Scientific, Orion Star A111). **(B)** Plot of pH value as a function of time. At t_0 = 0 min, the gas was switched from 100% N_2 to 90% N_2 and 10% CO_2. In unbuffered solution (*A. bidest*, black traces), the bulk pH dropped by approximately 1.7 units over the course of 25 minutes. No such acidification was observed with buffer solution (blue traces). These data show that 20 mM buffer is sufficient to keep the pH value of the protein solution constant in the presence of 10% CO_2 for at least 25 min. **(C)** In ATR FTIR spectroscopy, much smaller sample volumes are probed, typically 1–3 µl. Therefore, any direct kinetic comparison should be avoided. The figure shows a schematic depiction of the silicon crystal with a drop of liquid (yellow) or a protein film (green). **(D)** ATR FTIR difference spectra of *A. bidest* in the presence of 10% CO_2. No HCO_3^- bands are observed although a strong signal at 2343 cm^{-1} (CO_2 in aqueous solution) is visible. This is due to the acidification in pure water, as demonstrated in panel **(B)**. **(D)** ATR FTIR difference spectra of 25 mM Tris/HCl (pH 8) in the presence of 10% CO_2. While the signal at 2343 cm^{-1} indicates a similar concentration of CO_2, bands at 1620, 1360, and ~1300 cm^{-1} evidence residual HCO_3^- formation.

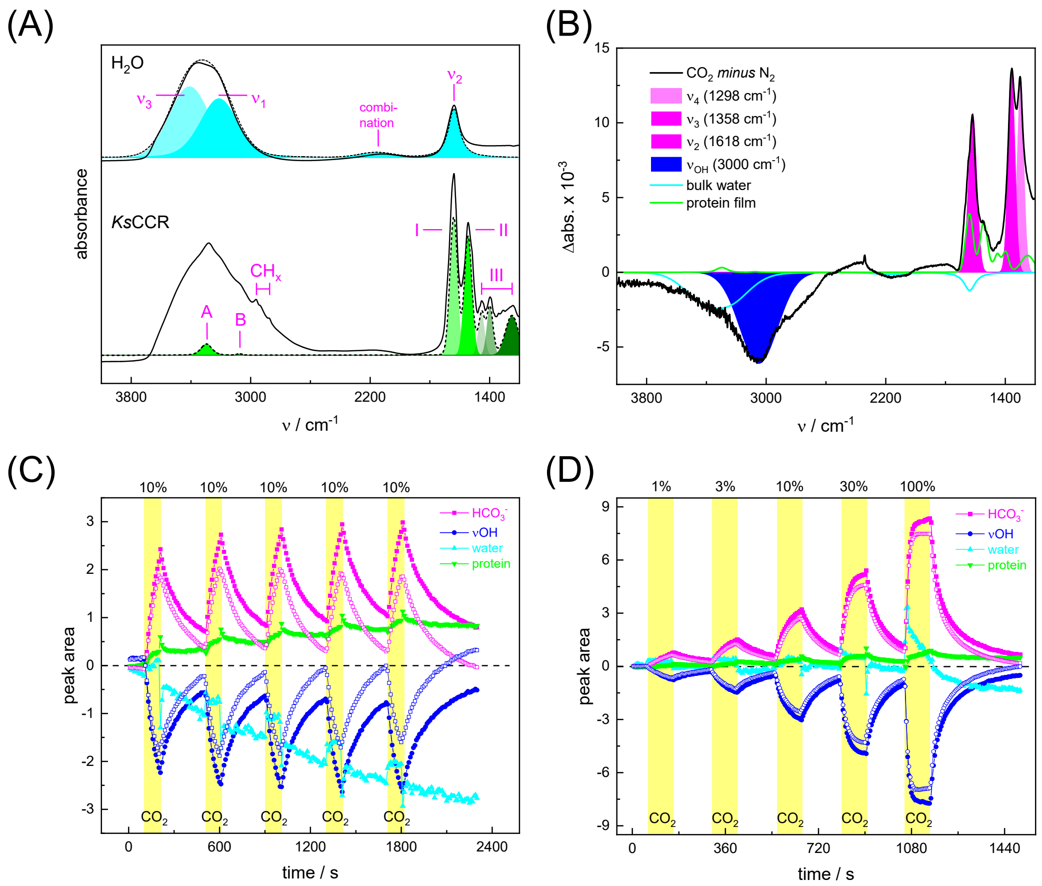

Figure S3. **(A)** FTIR absorbance spectra of liquid water (H_2O) and hydrated protein film (*Ks*Ccr). The H_2O spectrum comprises the asymmetric and symmetric OH stretching modes (ν_3, ν_1), the HOH bending mode (ν_2), and the so-called 'combination band' (cyan). The envelope is shown as a dashed line. The contributions of liquid water can be found in the spectrum of hydrated *Ks*Ccr as well; however, the fit is not shown for clarity. The protein spectrum comprises amide contributions A, B, I, II, and III (green). The envelope is shown as a dashed line. Note that the carbohydrate bands (CH_x) are not included in the fit. **(B)** Representative 'CO_2–N_2' FTIR difference spectrum of *Ks*Ccr, including the fits for unspecific changes, *i.e.*, a minor decrease of liquid water (cyan traces, negative) that is accompanied by a small increase of protein concentration (green traces, positive). Water and protein fits are derived from data in panel A. The accumulation of bicarbonate (magenta) in the film is followed *via* the sum of peak area including bands at 1298 cm^{-1} (ν_4), 1358 cm^{-1} and 1618 cm^{-1} (ν_2 and ν_3). The 'special' water band is centered at 3000 cm^{-1} (νOH, blue). **(C)** Plot of the peak area for bicarbonate, νOH, bulk water, and protein against time. The atmosphere above the hydrated *Ks*Ccr film is exchanged from 100% N_2 to 10% CO_2 (yellow area) in five consecutive steps, emphasizing the reversibility of the process. The peak area for bicarbonate and νOH is plotted 'as observed' (full symbols) and corrected for changes in protein concentration (open symbols). Due to the smaller spectral overlap, changes in humidity were neglected. **(D)** Plot of the peak area for bicarbonate, νOH, bulk water, and protein against time. The atmosphere above the hydrated *Ks*Ccr film is exchanged from 100% N_2 to 1–100% CO_2 (yellow area) in five consecutive steps, emphasizing the connection between band intensity and CO_2 partial pressure. The peak area for bicarbonate and νOH is plotted 'as observed' (full symbols) and corrected for changes in protein concentration (open symbols). Due to the smaller spectral overlap, changes in humidity were neglected.

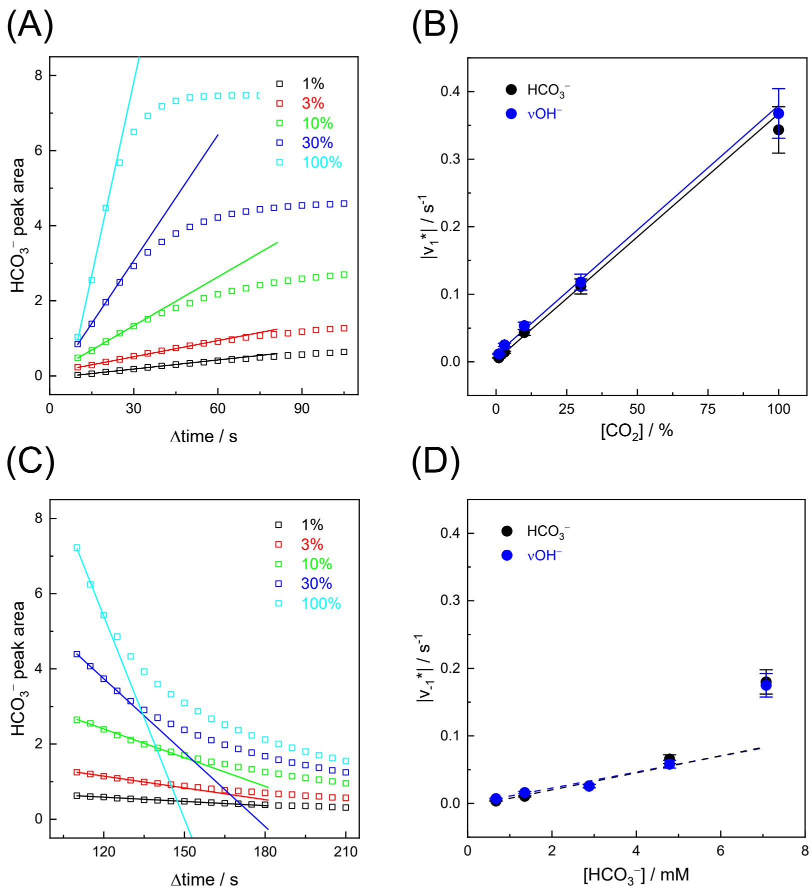

Figure S4. (A) Same data as in **Figure S3D**. The increase of the HCO_3^- signal under 1–100% is plotted against "Δtime" for visual comparison. The first three data points are fitted linearly to obtain the initial velocity v_1* of the CO_2 hydration reaction. **(B)** Initial velocity plotted against CO_2 concentration. The linear dependence suggests (pseudo-) first order kinetics. See **Figure S8** for the determination of experimental variation. **(C)** Same data as in **Figure S3D**. The decrease of the HCO_3^- signal after accumulation under 1–100% is plotted against "Δtime". The first three data points are fitted linearly to obtain the initial velocity v_{-1}* of the HCO_3^- dehydration reaction ($R^2 > 0.99$). **(D)** Initial velocity plotted against HCO_3^- concentration. The latter was obtained from reference experiments (**Figure S7**). The data do *not* agree with linear regression ($R^2 < 0.9$), which indicates a more complex reaction order. See **Figure S10** for the determination of experimental variation.

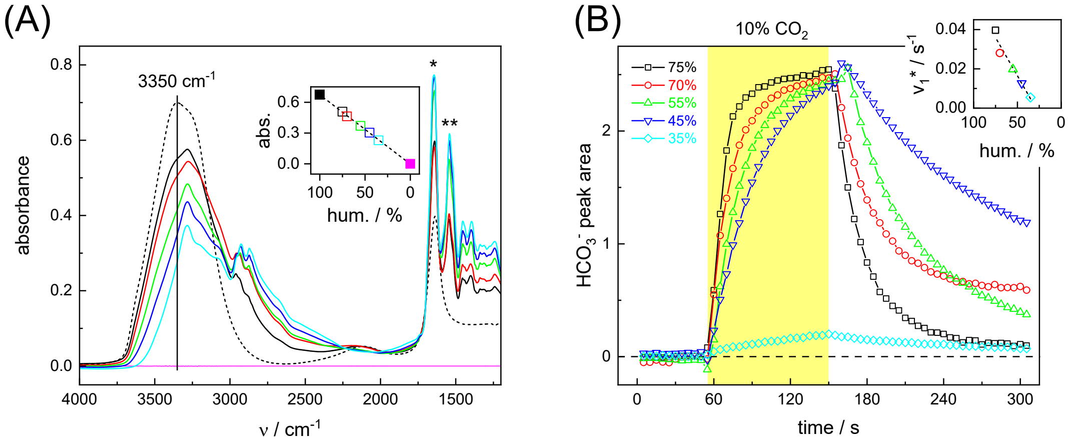

Figure S5. (A) FTIR spectra of liquid H_2O (dashed line), the empty ATR cell (magenta line) and a *Ks*Ccr protein films at five different levels of humidity. The humidity was adjusted by manually mixing water-free N_2 carrier gas ('dry') and N_2 aerosol ('wet'). All spectra show steady-state conditions after 5–10 min of equilibration. Inset: The 3350 cm^{-1} absorbance value for liquid H_2O (100%) and the empty cell (0%) define the calibration curve of water content that allows calculating the humidity level of the five *Ks*Ccr protein films (see legend in panel (B)). Note that the decrease in humidity is associated with an increase in protein concentration (* amide I, ** amide II). **(B)** CO_2 conversion kinetics of the five *Ks*Ccr protein films. The plot shows the HCO_3^- peak area as a function of time that increases in the presence of 10% CO_2 (yellow area) and decreases when the atmosphere is swept back to N_2. Between 75% and 45% water content, the initial reaction velocities decrease (inset) while the steady-state levels of HCO_3^- at t = 150 s are not significantly affected by the humidity level. Below 45% water the protein solution forms a 'cake' and becomes largely impenetrable to gas[7], resulting in a large decrease of CO_2 conversion activity. These data demonstrate that the observed kinetics are systematically affected by the humidity. A direct comparison with aqueous reaction assays is not advised. Carefully controlling the humidity level, however, different FTIR experiments can be compared both qualitatively and quantitative.

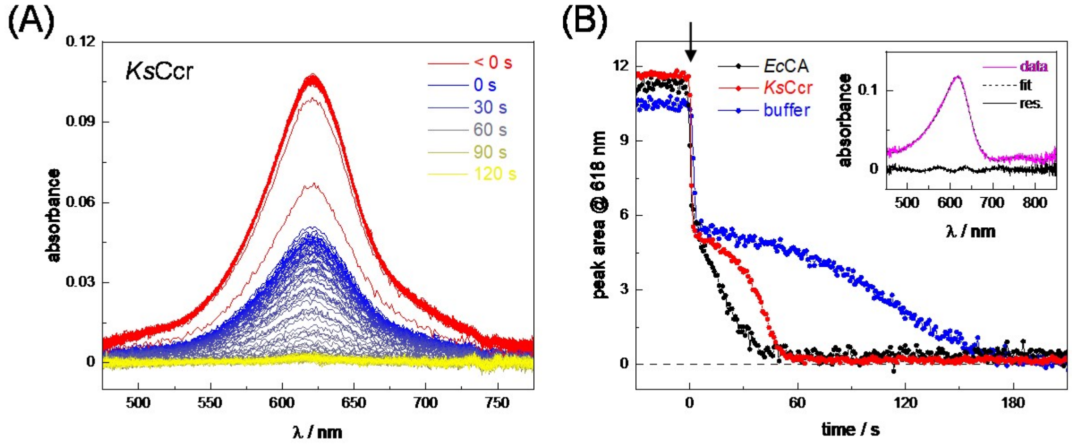

Figure S6. (A) Exemplary data set of CO_2 hydration *via* *Ks*Ccr as probed by UV/vis spectroscopy. The spectrum shows the decrease of the baseline-corrected 618 nm band of bromothylmol blue band at increasingly acidic pH. Here, the red spectra (t < 0 s) indicate an initial drop of intensity when the solution was diluted with carbonated buffer *ad* 1 ml. The spectral transition (blue to yellow) depicts the CO_2 hydration reaction between 0–120 s as catalyzed by *Ks*Ccr. **(B)** Time traces for the decrease of the 618 nm band of bromothylmol blue band for *Ec*CA (black), *Ks*Ccr (red), and buffer (blue). The inset shows how raw data was fitted with a single Lorentz function and a polynomial background. The arrow at t = 0 s marks the injection of carbonated buffer, leading to a drop in intensity due to dilution. CO_2 hydration contributed to the spectra afterwards. These data highlight (*i*) the superior activity of *Ec*CA and (*ii*) show that *Ks*Ccr catalyzes CO_2 hydration clearly faster than the buffer. See Experimental Methods for experimental details.

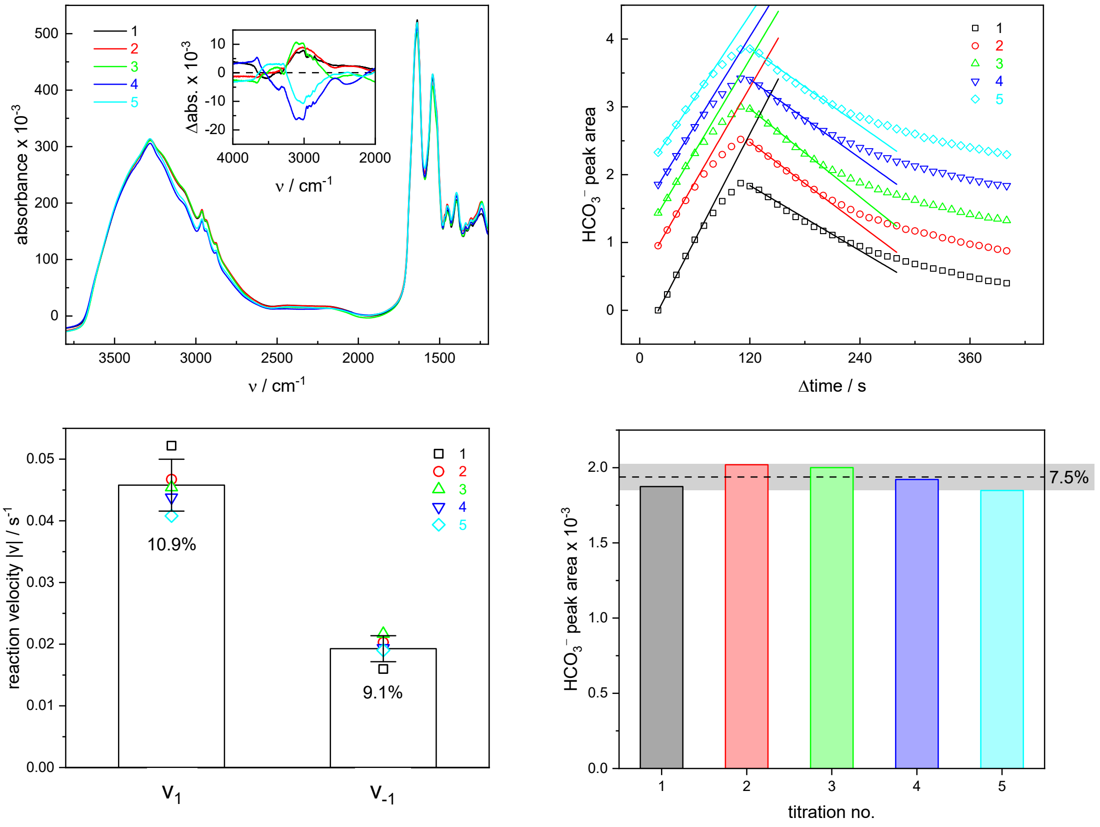

Figure S7. (A) Five films of *Ks*Ccr under N_2 at similar humidity. Inset: subtraction of the weighted averaged from each spectrum suggest less than 10% deviation in humidity. **(B)** The increase and decrease of bicarbonate over time from five *Ks*Ccr protein films in the presence and absence of 10% CO_2 is shown in a stacked plot. Linear fits of the first three data points for the $N_2 \rightarrow CO_2$ and $CO_2 \rightarrow N_2$ step yield apparent reaction velocities v_1* and v_{-1}*, respectively, that describe the velocity of the system to converge toward steady-state conditions. **(C)** According to these data, the rate constants are $v_1^* = 0.046\ s^{-1}$ (experimental deviation 10.9%) and $v_{-1}^* = 0.019\ s^{-1}$ (experimental deviation 9.1%). These values are used to compare data in **Figure 2D** of the main script. **(D)** The bicarbonate peak area at the end of each CO_2 step (dashed line in panel B) is shown. These data yield an experimental deviation of 7.5%, which is used to compare data in **Figure 4A** of the main script.

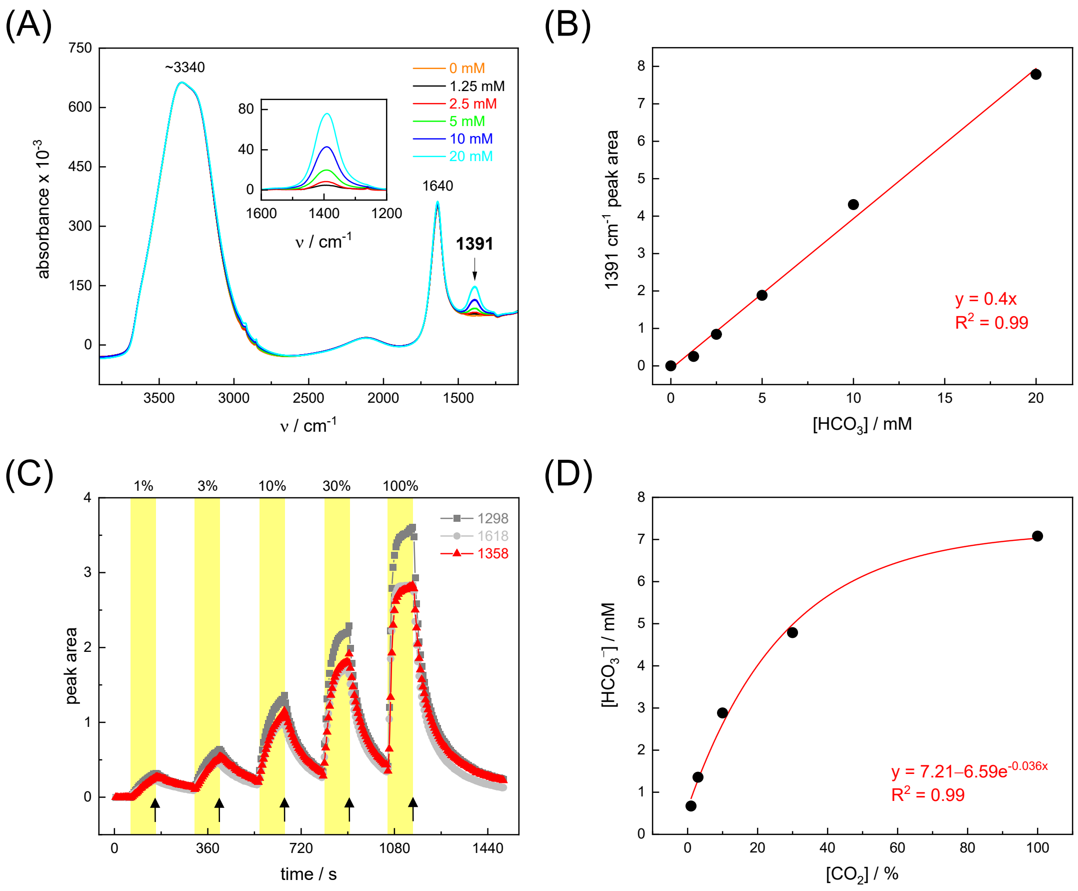

Figure S8. (A) FTIR absorbance spectra of Na_2CO_3 solution (0–20 mM) in water. The bands at ~3340 and 1640 cm^{-1} stem from liquid H_2O (see **Figure S3**), the band at 1391 cm^{-1} is assigned to the symmetric CO_2 stretching mode of bicarbonate (ν_3). The asymmetric CO_2 stretching frequency between 1630–1620 cm^{-1} (ν_2) is masked by the H_2O bending vibration. When the spectrum of pure water ('0 mM Na_2CO_3') is subtracted from the other signals, the ν_3 bicarbonate vibration appears as a distinct Gaussian band (inset) that can be fitted and used for calibration. **(B)** Based on the peak area of the band at 1391 cm^{-1} and the bicarbonate concentration of the solution, the data points suggest linear regression with a slope of y = 0.4x ($R^2 > 0.99$). **(C)** Same data as in **Figure S3D** (CO_2 titration of *Ks*Ccr) but here the three components of the bicarbonate signature are shown separately. The band at 1358 cm^{-1} (red traces) corresponds to the 1391 cm^{-1} signal of Na_2CO_3 and can be used for quantification using the formula in panel **(B)**. **(D)** Plotting the HCO_3^- concentration at the end of each CO_2-step (arrows in panel **(C)**) against CO_2 partial pressure, an exponential dependence becomes visible.

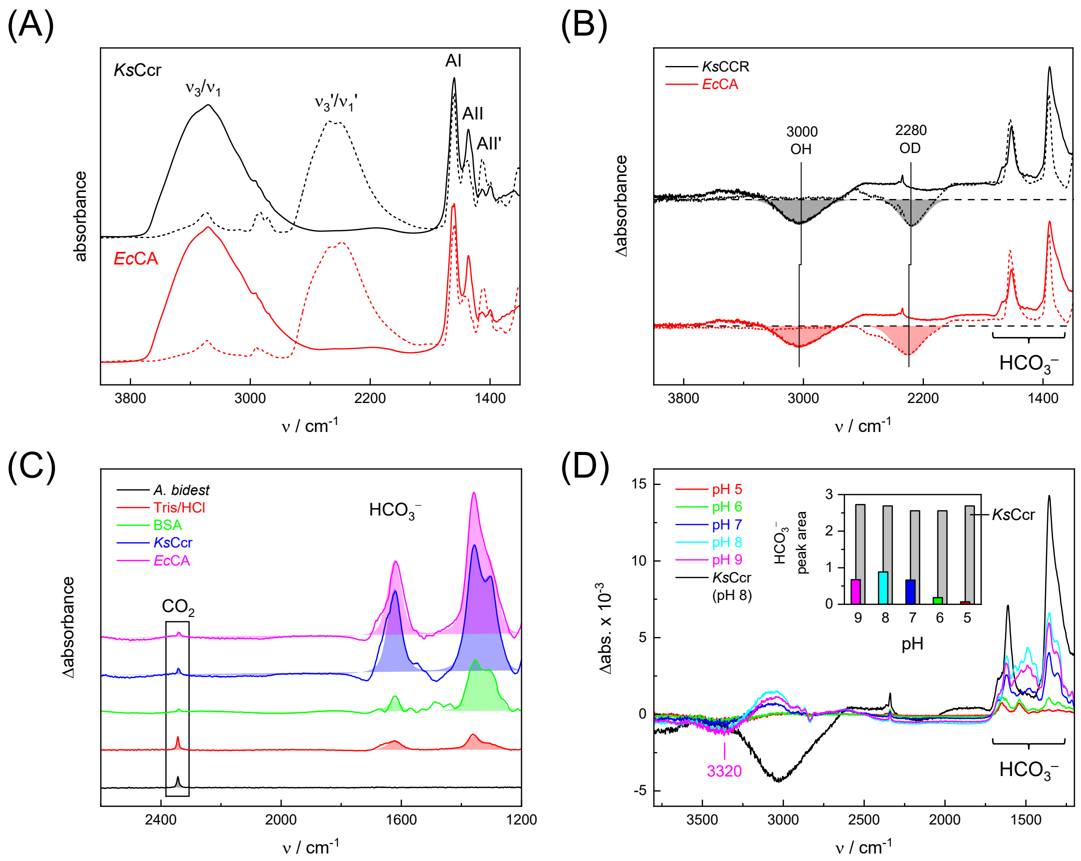

Figure S9. (A) Comparison of FTIR absorbance spectra from protein films of *Ks*Ccr and *Ec*CA under conditions of stable humidity with H_2O or D_2O (dashed traces). The overall similar amide band intensities (AI and AII/AII') and humidity level allows comparing the difference spectra of the reaction with CO_2 (see **Figure 4** in the main script). **(B)** 'CO_2–N_2' FTIR difference spectra of *Ks*Ccr (black) and *Ec*CA (red) in H_2O and D_2O (full and dashed lines, respectively). The data indicate bicarbonate formation and H_2O consumption by both enzymes. **(C)** Comparison of 'CO_2–N_2' spectra for water (black), Tris/HCl (red), BSA (green), *Ks*Ccr (blue), and EcCA (magenta) after 60 s in the presence of 10% CO_2. The peak area for CO_2 and HCO_3^- was determined as described in **Figure S3** (shaded area). Note the strong CO_2 band at 2343 cm^{-1} in water and buffer solution despite the low bicarbonate formation. **(D)** 'CO_2–N_2' FTIR difference spectra of *Ks*Ccr (black, pH 8) and BSA (colored traces, pH 5–9). BSA shows residual bicarbonate formation with a weak negative feature best fitted with the mean OH vibrations of bulk H_2O (v_1/v_3 at ~3320 cm^{-1}), indicating how BSA consumes bulk water upon CO_2 hydration. The spectra are most intense at pH 8 (cyan traces), hinting at optimal CO_2 hydration activity at alkaline pH, which is expected for the catalyst-free system.[25] Based on these data and similar experiments with *Ks*Ccr at pH 5–9, the inset plots the carbonate formation after 60 s. Note that CO_2 hydration with KsCcr is basically pH-independent in this pH range.

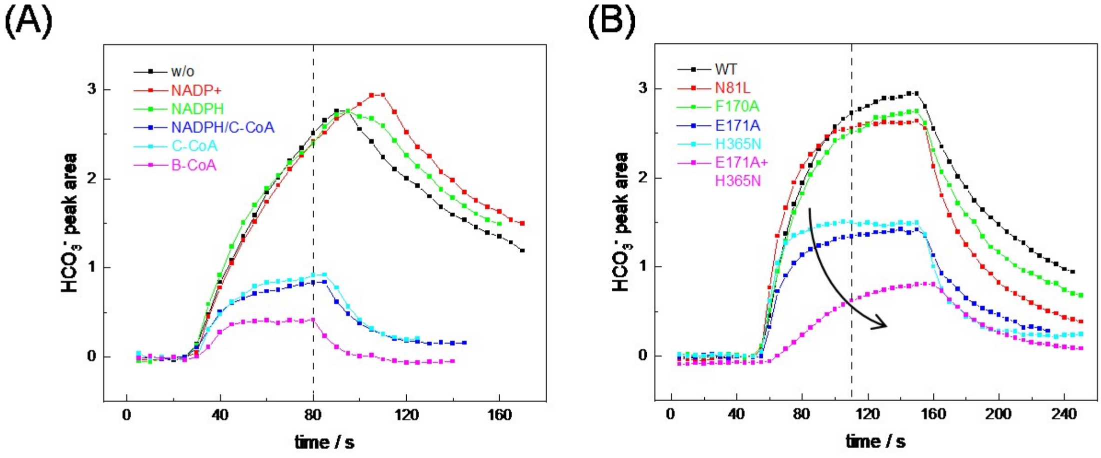

Figure S10. (A) Comparison of CA-like activity in *K*sCcr protein films under conditions of stable humidity in the presence of various substrates (B-CoA, C-CoA) and/or cofactors (NADPH, $NADP^+$). 60 s after addition of 10% CO_2 to the atmosphere (dashed line), the data facilitate a comparison among the different compositions (see **Figure 4** in the main script). **(B)** Comparison of CA-like activity in *K*sCcr protein films under conditions of stable humidity for wild-type *K*sCcr and five amino acid variants. 60 s after addition of 10% CO_2 to the atmosphere (dashed line), the data facilitate a comparison among the different variants (see **Figure 4** in the main script). The arrow highlights the decrease of CO_2 hydration velocity from *Ks*Ccr H365N (cyan) to E171A (blue) to H365N/E171A (magenta).

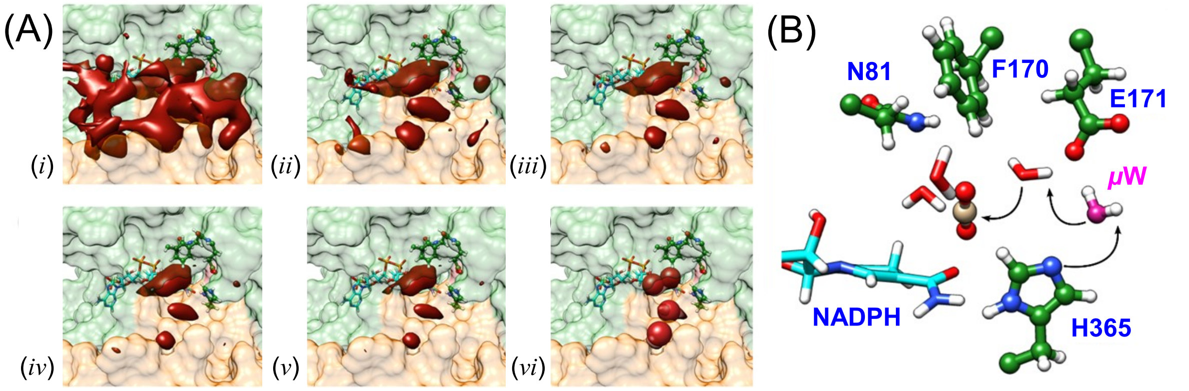

Figure S11. (A) The surface in red show regions in the active site where the CO_2 concentration is 2, 5, 10, 15, or 20 times larger than in the bulk obtained as time average from gromaρs, (i)–(v) respectively. The position of the binding sites in dark red spheres shown in the manuscript match the center of the 20 times bulk CO_2 concentration lobules (vi). **(B)** The nucleophilic attack of CO_2 in the active site *via* bridging water species μW may involve additional water moieties, according to MD simulations.

References

[1] G. M. M. Stoffel, D. A. Saez, H. DeMirci, B. Vögeli, Y. Rao, J. Zarzycki, Y. Yoshikuni, S. Wakatsuki, E. Vöhringer-Martinez, T. J. Erb, Proc. Natl. Acad. Sci. 2019, 201901471.

[2] M. Kitagawa, T. Ara, M. Arifuzzaman, T. Ioka-Nakamichi, E. Inamoto, H. Toyonaga, H. Mori, DNA Res. 2006, 12, 291–299.

[3] A. R. Oliveira, C. Mota, C. Mourato, R. M. Domingos, M. F. A. Santos, D. Gesto, B. Guigliarelli, T. Santos-Silva, M. J. Romão, I. A. Cardoso Pereira, ACS Catal. 2020, 10, 3844–3856.

[4] D. Peter, B. Vögeli, N. Cortina, T. Erb, Molecules 2016, 21, 517.

[5] B. Vögeli, K. Geyer, P. D. Gerlinger, S. Benkstein, N. S. Cortina, T. J. Erb, Cell Chem. Biol. 2018, 25, 833-839.e4.

[6] K. M. Wilbur, N. G. Anderson, J. Biol. Chem. 1948, 176, 147–154

[7] S. T. Stripp, ACS Catal. 2021, 11, 7845–7862.

[8] V. Gapsys, B. L. de Groot, J. Chem. Inf. Model. 2017, 57, 109–114.

[9] V. Gapsys, A. Yildirim, M. Aldeghi, Y. Khalak, D. van der Spoel, B. L. de Groot, Commun. Chem. 2021, 4, 61.

[10] M. J. Abraham, T. Murtola, R. Schulz, S. Páll, J. C. Smith, B. Hess, E. Lindahl, SoftwareX 2015, 1–2, 19–25.

[11] J. Huang, S. Rauscher, G. Nawrocki, T. Ran, M. Feig, B. L. de Groot, H. Grubmüller, A. D. MacKerell, Nat. Methods 2017, 14, 71–73.

[12] S. Boonstra, P. R. Onck, E. van der Giessen, J. Phys. Chem. B 2016, 120, 3692–3698.

[13] K. Vanommeslaeghe, E. Hatcher, C. Acharya, S. Kundu, S. Zhong, J. Shim, E. Darian, O. Guvench, P. Lopes, I. Vorobyov, A. D. Mackerell Jr., J. Comput. Chem. 2010, 31, 671–690.

[14] K. Vanommeslaeghe, A. D. MacKerell, J. Chem. Inf. Model. 2012, 52, 3144–3154.

[15] J. J. Pavelites, J. Gao, P. A. Bash, A. D. Mackerell Jr., J. Comput. Chem. 1997, 18, 221–239.

[16] A. Aleksandrov, M. Field, Phys. Chem. Chem. Phys. 2011, 13, 10503.

[17] G. Bussi, D. Donadio, M. Parrinello, J. Chem. Phys. 2007, 126, 014101.

[18] H. J. C. Berendsen, J. P. M. Postma, W. F. van Gunsteren, A. DiNola, J. R. Haak, J. Chem. Phys. 1984, 81, 3684–3690.

[19] T. Darden, D. York, L. Pedersen, J. Chem. Phys. 1993, 98, 10089–10092.

[20] C. H. Bennett, J. Comput. Phys. 1976, 22, 245–268.

[21] M. H. M. Olsson, C. R. Søndergaard, M. Rostkowski, J. H. Jensen, J. Chem. Theory Comput. 2011, 7, 525–537.

[22] R. Briones, C. Blau, C. Kutzner, B. L. de Groot, C. Aponte-Santamaría, Biophys. J. 2019, 116, 4–11.

[23] M. Meneghello, A. R. Oliveira, A. Jacq-Bailly, I. A. C. Pereira, C. Leger, V. Fourmond, Angew. Chem. Int. Ed. 2021, 60, 9964–9967.

[24] X. Yu, D. Niks, A. Mulchandani, R. Hille, J. Biol. Chem. 2017, 292, 16872–16879.

[25] J. N. Butler, Carbon Dioxide Equilibria and Their Applications, Routledge, New York, 1991.
